# High-urgency heart transplantation and outcome trade-offs: early post-transplant infection and mortality

**DOI:** 10.1093/eschf/xvag167

**Published:** 2026-06-10

**Authors:** Kyu-Sun Lee, Darae Kim, Jin-Oh Choi, Hae-Young Lee, Myoung Soo Kim, Hyungseop Kim, Dong-Ju Choi, Sang Eun Lee, Seok-Min Kang, Soo Yong Lee, Hyun-Jai Cho, KOTRY study group, Cheol Kim, Jaewon Oh, Jin Joo Park, Min Ho Ju, Sung Ho Jung, Jung Ae Hong, Jeehoon Kang, Jeongsu Kim, Junho Hyun, Mi Hee Lim, Minjae Yoon, Nam Hee Park, Sun Hwa Lee

**Affiliations:** Department of Internal Medicine and Division of Cardiology, Eulji University Hospital and Eulji University School of Medicine, Daejeon, Republic of Korea; Department of Internal Medicine, Samsung Medical Center, Sungkyunkwan University College of Medicine, Seoul, Republic of Korea; Department of Internal Medicine, Samsung Medical Center, Sungkyunkwan University College of Medicine, Seoul, Republic of Korea; Department of Internal Medicine, Seoul National University Hospital and Seoul National University College of Medicine, Seoul, Republic of Korea; Department of Surgery, Yonsei University College of Medicine, Seoul, Republic of Korea; Division of Cardiology, Keimyung University Dongsan Medical Center, Daegu, Republic of Korea; Cardiovascular Center, Department of Internal Medicine, Seoul National University Bundang Hospital, Seoul National University College of Medicine, Seongnam, Korea; Department of Internal Medicine, Asan Medical Center, University of Ulsan College of Medicine, Seoul, Republic of Korea; Department of Internal Medicine, Yonsei University College of Medicine, Seoul, Republic of Korea; Division of Cardiology, Department of Internal Medicine, Pusan National University Yangsan Hospital, Yangsan, Republic of Korea; Department of Internal Medicine, Seoul National University Hospital and Seoul National University College of Medicine, Seoul, Republic of Korea

**Keywords:** Heart transplantation, Urgency status, Post-transplant infections, Mortality, Korean Organ Transplant Registry

## Abstract

**Background and Aims:**

To evaluate differences in post-transplant outcomes by pre-transplant urgency status, focusing on early post-transplant infection and its contribution to mortality.

**Methods:**

We retrospectively analysed 801 adult heart transplant recipients enrolled in the Korea Organ Transplant Registry (April 2014–December 2021). Recipients were classified as Status 0 (highest urgency; *n* = 287) and Status 1–3 (*n* = 514). Outcomes included all-cause mortality, post-transplant infection, acute allograft rejection, and cardiac allograft vasculopathy (CAV) up to 5 years. Mediation, landmark, and multivariable time-dependent Cox models assessed the impact and determinants of infection.

**Results:**

During 5-year follow-up, 128 recipients (16.0%) died. Status 0 recipients had higher mortality than Status 1–3 recipients (28.0% vs. 13.5%; *P* < .001). Infection was the leading cause of death and was more frequent in Status 0 recipients at 1 and 6 months, whereas rejection and CAV rates were similar between groups. Infection at 1, 6, and 12 months was strongly associated with mortality and mediated the association between urgency status and mortality, accounting for 47.4%, 34.9%, and 34.2% of total effect, respectively. After adjustment for pre- and post-transplant organ support, urgency status was no longer independently associated with infection risk, whereas prolonged mechanical ventilation (>24 h) remained the strongest predictor.

**Conclusion:**

Status 0 heart transplant recipients had higher mortality and early post-transplant infection risk than Status 1–3 recipients. Early infection, largely associated with greater clinical severity and prolonged mechanical ventilation, may explain the excess mortality in this high-urgency group.

## Graphical Abstract

**Graphical Abstract:**
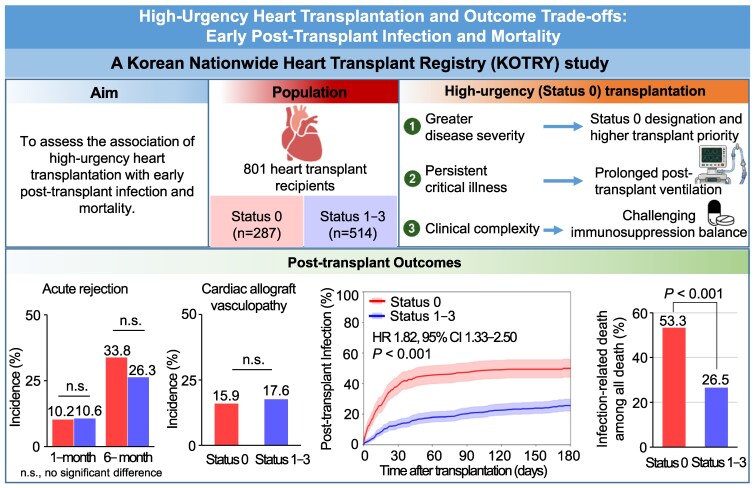
In this nationwide Korean Organ Transplant Registry (KOTRY) cohort of 801 heart transplant recipients, Status 0 recipients exhibited a substantially higher burden of advanced heart failure, comorbidities, and invasive life-support therapies. Although the incidence of acute allograft rejection and cardiac allograft vasculopathy were comparable between urgency groups, Status 0 recipients experienced significantly higher rates of early post-transplant infection, which was strongly associated with increased mortality.

## Introduction

Heart transplantation remains the definitive treatment option for patients with end-stage or advanced heart failure, and the global demand for donor hearts continues to increase, including in South Korea.^1,2^ However, despite advances in medical therapy and mechanical circulatory support (MCS), the persistent shortage of donor organs has resulted in prolonged waiting times and substantial waitlist mortality, underscoring the need for effective and equitable organ allocation strategies.^3,4^ Accordingly, heart transplant allocation systems are designed to prioritize candidates based on clinical urgency while also optimizing overall transplant outcomes.^4^ However, their structure, granularity, and prioritization criteria vary across countries and regions.

In South Korea, heart transplantation is governed by the Korean Network for Organ Sharing (KONOS), a centralized national system using a four-tier urgency-based classification, with Status 0 representing the highest priority. Status 0 typically includes patients requiring extracorporeal membrane oxygenation (ECMO), mechanical ventilation, or other forms of advanced life support, whereas Status 1–3 represent progressively lower levels of medical urgency. In contrast, the United Network for Organ Sharing (UNOS) in the USA uses a more granular six-tier system (Status 1–6) that incorporates detailed stratification based on device support and physiological severity.^4^ Eurotransplant uses categories such as high urgency, approved combined organ, transplantable, and not transplantable (NT), thereby incorporating not only medical severity but also transplantability.^5^ Beyond these structural differences, allocation policies have also evolved over time within each system. In South Korea, the current four-tier allocation system has been in place since 2000, with candidates classified from Status 0, representing the highest priority, to Status 3, while Status 7 indicates temporary inactive status.^2^ A policy revision in 2017 reclassified selected high-acuity Status 1 patients into Status 0, while the overall framework of the Korean allocation system remained unchanged. In the USA, the heart allocation policy was revised in 2018, replacing the former three-tier system with a six-tier framework aimed at improving prioritization of critically ill patients, reducing waiting times for high-acuity candidates, and incorporating more objective clinical criteria.^6^ Subsequent studies have demonstrated improved access to transplantation for the sickest patients and reductions in waitlist mortality following these policy changes.^6,7^ Although heart transplant allocation systems differ in structure, granularity, and prioritization, they share several fundamental principles. Across Korea, USA, and Europe, these systems prioritize medical urgency, incorporate objective clinical criteria to stratify candidates, consider waiting time as an important secondary factor, and aim to promote fairness and equity while improving both waitlist and post-transplant outcomes.

However, the extent to which urgency-based allocation affects post-transplant outcomes remains incompletely understood, particularly in the context of evolving allocation policies and improved waitlist outcomes. In South Korea, it remains unclear whether short- and long-term post-transplant outcomes differ between Status 0 and Status 1–3 recipients under the KONOS urgency-based heart allocation system. Given that the Korean allocation system differs substantially from both Eurotransplant and UNOS in urgency stratification and waiting-list structure, findings from Western cohorts may not be directly generalizable to Korean recipients. This issue is particularly relevant for Status 0 recipients, who frequently require prolonged intensive care, temporary MCS, and invasive devices before transplantation. Such pre-transplant exposures may increase vulnerability to early post-transplant complications, highlighting the importance of understanding downstream consequences associated with high-urgency transplantation. Infection, acute rejection, and cardiac allograft vasculopathy (CAV) are well-recognized determinants of post-transplant prognosis.^8–10^ However, whether these outcomes vary according to pre-transplant urgency status within allocation systems has not been comprehensively evaluated. In particular, it remains unclear whether early post-transplant infection disproportionately affects high-urgency recipients and contributes to reduced survival outcomes, representing a critical outcome trade-off of allocation-driven high-urgency transplantation.

Accordingly, this study aimed to evaluate allocation-driven differences in short- and long-term post-transplant outcomes according to pre-transplant urgency status as defined by KONOS, using data from the Korean Organ Transplantation Registry (KOTRY). We focused particularly on the incidence and prognostic impact of early post-transplant infection and its contribution to mortality to better characterize the outcome trade-offs associated with high-urgency heart transplantation.

## Methods

### Data source and study population

The KOTRY, established in 2014, is the first nationwide prospective cohort of solid organ transplantation in Korea. Heart transplant recipients were consecutively enrolled from multiple tertiary centres after providing written informed consent. Data were collected using standardized protocols, and detailed registry methodology has been previously described.^11,12^ Between April 2014 and December 2021, a total of 813 adult heart transplant recipients were enrolled in the KOTRY heart transplant cohort. After excluding 12 recipients due to study withdrawal or loss to follow-up, 801 recipients were included in the final analysis. Medical urgency on the heart transplant waitlist was determined based on listing status as defined by the four-tier allocation system of the KONOS, by which candidates were stratified from the highest to lowest urgency. In this study, recipients were classified into two groups according to pre-transplant urgency status at the time of transplantation: Status 0 (highest urgency) and Status 1–3. The criteria defining each urgency status are summarized in Supplementary Table S1. This study was conducted in accordance with the principles of the Declaration of Helsinki and institutional guidelines. The study protocol was reviewed and approved by the Institutional Review Board of Seoul National University Hospital (IRB No. 1406-082-588). Written informed consent was obtained from all participants or their legally authorized representatives prior to enrolment in the KOTRY. The privacy rights of all human subjects were protected, and all data were anonymized prior to analysis.

### Study outcomes

The primary outcome was all-cause mortality. Secondary outcomes included post-transplant infections, acute allograft rejection, and CAV. Outcomes were assessed at early post-transplant intervals of 1, 6, and 12 months and annually up to 5 years after transplantation, according to the KOTRY heart transplant cohort protocol.

After transplantation, the cause of death was ascertained according to the circumstances of death. For deaths occurring during hospitalization, the primary cause of death was adjudicated by the attending transplant physician based on clinical information, laboratory findings, imaging studies, and the overall clinical course. For out-of-hospital deaths, the cause of death was obtained from the national death registry linked to KOTRY enrolment records. Causes of death were categorized according to pre-specified criteria in the KOTRY heart transplant cohort protocol as cardiovascular disease, sudden cardiac death, infection, acute rejection, liver disease, malignancy, suicide, accident, multi-organ failure/disseminated intravascular coagulation, respiratory failure, cerebrovascular disease, bleeding, other, or unknown. If the cause of death did not fit any pre-defined category, it was classified as ‘other’, and the specific cause was additionally documented. If a definitive cause of death could not be established, it was classified as ‘unknown’. For all death events, both the date and cause of death were recorded in the electronic case report form.

For rejection surveillance, endomyocardial biopsy (EMB) was performed according to centre-specific protocols, with the KOTRY protocol recommending EMB at 30 days and at 3, 6, 12, and 24 months after heart transplantation. Routine EMB was generally discontinued after 24 months, with additional biopsies performed when clinically indicated, particularly when allograft rejection was suspected. Acute allograft rejection was classified according to International Society for Heart and Lung Transplantation (ISHLT) guidelines^13^ and categorized into acute cellular rejection (ACR) and acute antibody-mediated rejection (AMR). ACR was graded as 1R, 2R, 3R, or unspecified, and AMR was pathologically classified as AMR 1–3. Only biopsy-confirmed rejection cases were included in the analysis.

Post-transplant infections were defined based on the following criteria: (i) the presence of clinical signs or symptoms indicative of infection; (ii) the detection of pathogens, such as viruses, bacteria, or fungi, through laboratory testing (e.g. blood culture) or imaging evidence of an infectious focus; and (iii) infections requiring hospitalization or a treatment duration of at least 2 weeks.

CAV was diagnosed primarily by invasive coronary angiography (CAG), with intravascular ultrasound used as an adjunct when appropriate and coronary computed tomography angiography (CCTA) was used for follow-up evaluation when appropriate. According to the KOTRY protocol, routine CAG was performed 12 months after transplantation, followed by annual coronary evaluation using either CAG or CCTA. Additional coronary evaluation was performed whenever CAV was clinically suspected. CAV severity was classified as CAV 0 (insignificant), CAV 1 (mild), CAV 2 (moderate), or CAV 3 (severe) according to the ISHLT CAV grading system.

### Statistical analysis

Descriptive statistics were used to summarize the baseline characteristics and comorbidities of the study population. Categorical variables are presented as frequencies and percentages, while continuous variables are presented as mean ± standard deviation or median with inter-quartile range, depending on data distribution. Group comparisons were conducted using the χ^2^ test or Fisher’s exact test for categorical variables. Continuous variables were compared using the Student’s *t*-test or Wilcoxon rank-sum test for two-group comparisons and the Kruskal–Wallis test for comparisons involving more than two groups, as appropriate. The Cox proportional hazards regression model was used to estimate the risks of clinical outcomes, including all-cause mortality, post-transplant infection, acute allograft rejection, and CAV, and to identify associated risk factors. Hazard ratios (HRs) and 95% confidence intervals (CIs) were estimated using univariable and multivariable Cox regression models. Multivariable models were adjusted for clinically relevant covariates and variables that differed between urgency status groups at baseline, including age, sex, body mass index, diabetes mellitus, hypertension, chronic kidney disease, cause of transplantation, operation time, cold ischaemic time, natriuretic peptide level, and calculated panel-reactive antibody levels. These covariates were selected based on clinical relevance and/or statistically significant differences between urgency status groups at baseline.

The proportional hazards assumption was assessed using the Schoenfeld residuals. When a significant violation of the proportional hazards assumption was detected (*P* < .05), time-varying effects were modelled by including interaction terms between the relevant covariates and log(time). Kaplan–Meier curves were generated to describe time-to-event outcomes, including all-cause mortality, post-transplant infection, acute allograft rejection, and CAV, and between-group differences assessed using the log-rank test.

Causal mediation analysis was conducted to assess whether early post-transplant infection mediated the association between urgency status and mortality.^14–16^ A regression-based approach with non-parametric bootstrapping with 1000 iterations was used to estimate direct, indirect, and total effects, along with the 95% CIs and *P*-values. All statistical tests were two-sided, and a *P*-value of <.05 was considered statistically significant. Statistical analyses were performed using SPSS Statistics version 25.0 (IBM, Armonk, NY, USA) and Python 3.10.

## Results

### Urgency-based classification and baseline characteristics of recipients

Among 801 recipients, 287 (35.8%) underwent heart transplantation as Status 0, and 514 (64.2%) as Status 1–3 (Supplementary Figure S1). Baseline characteristics by pre-transplant urgency status are summarized in *Table 1*. The median age at transplantation was 56 years, and 70.2% were male. Diabetes mellitus, hypertension, and chronic kidney disease were present in 30.6%, 33.0%, and 18.4% of recipients, respectively. Cardiomyopathy (56.3%) and ischaemic heart disease (22.2%) were the most common overall indications for heart transplantation. Compared with Status 1–3, Status 0 recipients more frequently had diabetes mellitus, chronic kidney disease, and panel-reactive antibody levels >50%. They also had lower left ventricular ejection fraction, haemoglobin, and platelet counts, but higher natriuretic peptide levels at the time of transplantation. In Status 0 recipients, ischaemic heart disease and acute myocarditis were more common indications for heart transplantation, whereas cardiomyopathy predominated among Status 1–3 recipients. Status 0 recipients had shorter waiting time from transplant registration to transplantation, while operative, cold ischaemic, and warm ischaemic times were similar between groups. At discharge, Status 0 recipients showed higher tacrolimus trough levels and received lower steroid doses than Status 1–3 recipients.

**Table 1:** Baseline characteristics of heart transplant recipients based on urgency status

Variables	Overall (*n* = 801)	The highest priority status (Status 0) (*n* = 287)	Lower urgency status (Status 1–3) (*n* = 514)	*P*-value
Age (years)	56 (46–63)	55 (44–63)	57 (47–63)	.020
Sex (male)-no. (%)	562 (70.2)	203 (70.7)	359 (69.8)	.810
BMI (kg/m^2^)	22.3 (20.1–24.7)	22.5(19.9–25.0)	22.3 (20.1–24.5)	.362
Diabetes mellitus-no. (%)	245 (30.6)	104 (36.2)	141 (27.4)	.011
Hypertension-no. (%)	264 (33.0)	89 (31.0)	175 (34.0)	.390
Smoking status-no. (%)				.003
Never	466 (58.2)	171 (59.6)	295 (57.4)	
Current	84 (10.5)	43 (15.0)	41 (8.0)	
Former	241 (30.1)	69 (24.0)	172 (33.5)	
Previous cancer-no. (%)	61 (7.6)	19 (6.6)	42 (8.2)	.448
Chronic kidney disease. (%)	147 (18.4)	63 (22.0)	84 (16.4)	.057
Causes of transplant-no. (%)				
Ischaemic	178 (22.2)	80 (27.9)	98 (19.1)	.005
Cardiomyopathy	451 (56.3)	131 (45.6)	320 (62.3)	<.001
Valvular heart disease	32 (4.0)	9 (3.1)	23 (4.5)	.453
Myocarditis	28 (3.5)	27 (9.4)	1 (0.2)	<.001
Infiltrative disease	29 (3.6)	3 (1.0)	26 (5.1)	.003
Chemotherapy-induced	8 (1.0)	3 (1.0)	5 (1.0)	1.000
Panel-reactive antibody >50%				.002
Class I	91 (11.6)	48 (17.1)	43 (8.5)	
Class II	105 (13.4)	48 (17.1)	57 (11.4)	
Class I and II	42 (5.3)	25 (8.8)	17 (3.3)	
Donor-specific antibodies (+)	103 (12.8)	35 (13.5)	68 (12.5)	.489
LVEF (%)^a^	23.0 (18.0–30.0)	15.0 (11.6–19.4)	28.0 (21.0–32.5)	<.001
Lab findings^a^				
WBC (×10^3^/µl)	7.0 (5.3–9.4)	8.5 (6.5–13.1)	5.5 (4.9–7.2)	<.001
Haemoglobin (g/dl)	10.9 (9.3–12.6)	9.0 (8.1–9.9)	11.8 (10.8–13.2)	<.001
Platelet (×10^3^/µl)	158.0 (106.3–212.0)	67.0 (46.0–106.0)	188.0 (160.3–246.8)	<.001
BUN (mg/dl)	20.7 (15.4–31.0)	22.8 (17.1–31.5)	17.5 (14.0–34.0)	.001
Creatinine (mg/dl)	1.0 (0.8–1.4)	0.8 (0.6–1.4)	1.1 (0.7–1.2)	.547
BNP (pg/ml)	736.5 (342.8–1710.8)	1890.0 (1127.5–3874.0)	696.0 (393.0–2178.0)	<.001
NT-proBNP (pg/ml)	3808.0 (1638.5–9649.0)	4600.0 (2826.0–17 831.0)	3612.0 (786.0–4669.3)	<.001
Time from registration to transplant (days)	221.0 ± 486.0	136.0 ± 361.0	269.0 ± 537.0	<.001
Operation time (min)	342.0 (288.5–410.0)	346.0 (300.0–402.0)	333.5 (280.3–408.8)	.161
Cold ischaemic time (min)	95.0 (66.0–159.0)	93.0 (67.0–159.0)	96.0 (65.3–160.8)	.887
Warm ischaemic time (min)	52.0 (38.0–76.0)	52.0 (38.0–70.0)	50.5 (37.0–76.0)	.796
Immunosuppressants^b^				
Tacrolimus (ng/ml)	8.6 (6.9–10.2)	7.3 (4.2–9.0)	6.8 (4.7–9.0)	.003
MMF (mg)	1000 (1000–2000)	1000 (540–1440)	1000 (500–1440)	.136
Steroid (mg)	12.5 (10.0–25.0)	7.5 (6.3–10.0)	10.0 (7.5–10.0)	<.001
Everolimus (ng/ml)	2.9 (2.0–4.3)	2.0 (2.0–3.1)	2.0 (2.0–3.0)	.315

Abbreviations: BMI, body mass index; LVEF, left ventricular ejection fraction; WBC, white blood cell; BUN, blood urea nitrogen; BNP, b-type natriuretic peptide; NT-proBNP, n-terminal proBNP; MMF, mycophenolate mofetil

^a^Values measured at the time of transplantation

^b^Prescribed doses of MMF and steroids and serum levels of tacrolimus and everolimus at discharge

### Post-transplant outcomes by pre-transplant urgency status: mortality, causes of death, and incidence of infection, rejection, and CAV

**Figure 1:**
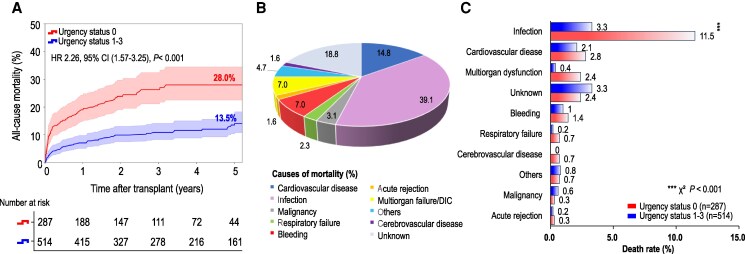
Five-year post-transplant mortality and causes of death according to urgency status. (*A*) Kaplan–Meier curves comparing all-cause mortality over 5 years after heart transplantation between Status 0 and Status 1–3 recipients. (*B*) The pie chart shows the distribution of causes of death among heart transplant recipients during the 5-year follow-up period. (*C*) The bar graph compares the distribution of causes of death between Status 0 and Status 1–3 recipients during the 5-year follow-up period. Abbreviations: HR, hazard ratio; CI, confidence interval

**Figure 2:**
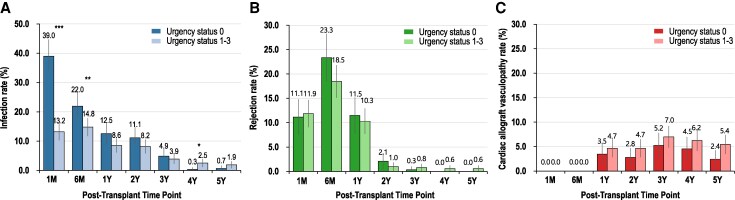
Temporal patterns of post-transplant infection, rejection, and cardiac allograft vasculopathy according to urgency status. Bar graphs showing the proportions of (*A*) post-transplant infection, (*B*) acute allograft rejection, and (*C*) cardiac allograft vasculopathy during the 5-year follow-up period according to urgency status. Asterisks indicate the level of statistical significance: *P < .05, **P < .01, ***P < .001

**Figure 3:**
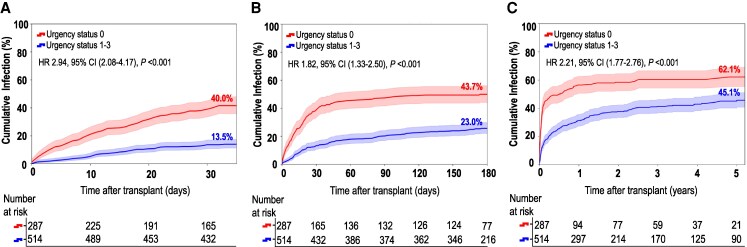
Post-transplant infection risk at early term and long-term follow-up by urgency status. Kaplan–Meier curves showing post-transplant infection risk according to urgency status at (*A*) 1 month, (*B*) 6 months, and (*C*) 5 years after heart transplantation. Abbreviations: HR, hazard ratio; CI, confidence interval

Of the 801 recipients, 128 (16.0%) died during the 5-year follow-up. Status 0 recipients had a significantly higher all-cause mortality risk than those with Status 1–3 (*Figure 1A*). The leading causes of death were infection (39.1%), cardiovascular disease (14.8%), multi-organ failure (7.0%), and post-operative bleeding (7.0%), with smaller proportions from malignancy (3.1%), respiratory failure (2.3%), acute rejection (1.6%), cerebrovascular disease (1.6%), and other causes (4.7%). However, 18.8% remained unspecified (*Figure 1B*). Deaths from infection and multi-organ failure were more frequent in Status 0 than Status 1–3 (*Figure 1C*). Post-transplant infection, rejection, and CAV according to urgency status are shown in *Figure 2*. Infection rates at 1 and 6 months were significantly higher in Status 0 recipients than in Status 1–3 (*Figure 2A*), whereas rejection and CAV rates showed no significant group differences (*Figure 2B* and *C*). The cumulative incidence of infection over time was consistently higher in Status 0, with the difference evident from the first month post-transplant and persisting over 5 years (*Figure 3*). In contrast, the risk of post-transplant rejection was similar at 1 month and numerically higher at 6 months in Status 0 recipients, but without statistical significance (Supplementary Figure S2A and S2B). These patterns remained unchanged in the additional analysis using the pre-revision urgency classification before the 2017 KONOS policy revision. In this sensitivity analysis, Status 0 recipients continued to show higher mortality and infection rates than Status 1–3 recipients, whereas rejection and CAV rates remained similar between groups (Supplementary Figure S3).

### Early post-transplant infection mediates the association between urgency status and mortality

**Figure 4:**
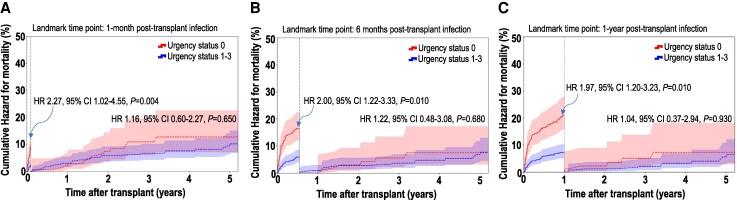
Landmark analysis of all-cause mortality according to urgency status among infection-free recipients. Kaplan–Meier curves comparing all-cause mortality between Status 0 and Status 1–3 recipients among patients who were alive and free of post-transplant infection at each landmark time point: (*A*) 1 month, (*B*) 6 months, and (*C*) 1 year after transplantation. Vertical dashed lines indicate the respective landmark time points. Mortality comparisons between urgency status groups were performed using Cox proportional hazards models starting from each landmark time point. Abbreviations: HR, hazard ratio; CI, confidence interval

Among post-transplant complications, only infection was significantly associated with mortality. In particular, post-transplant infection at 1, 6, and 12 months was significantly associated with an increased risk of death (Supplementary Table S2 and Supplementary Figure S4). Mediation analysis showed that infection significantly mediated the association between urgency status and mortality at all three time points, accounting for 47.4%, 34.9%, and 34.2% of the total effect on mortality, respectively (Supplementary Figure S5). Landmark analysis further supported this finding. Before each landmark time point, Status 0 recipients had significantly higher mortality than Status 1–3 recipients. However, after excluding patients who had developed post-transplant infection before each respective landmark time point, the mortality difference between the groups was no longer statistically significant (*Figure 4*). To determine whether the effect of urgency status on post-transplant infection risk changed over time, an extended Cox model incorporating a time-dependent interaction term was additionally applied. This analysis revealed that Status 0 recipients had a significantly higher risk of infection during the early post-transplant period and that this excess risk gradually declined over time (Supplementary Table S3). These findings indicate that the effect of urgency status on infection risk was time-varying rather than constant throughout the follow-up period. Taken together, these findings suggest that the higher mortality observed in Status 0 recipients was largely explained by increased susceptibility to early post-transplant infection rather than by urgency status itself.

### Pre- and post-transplant organ support is associated with increased infection risk in Status 0 recipients

During the waiting period, Status 0 recipients more frequently received MCS than Status 1–3 recipients (90.2% vs. 13.0%, *P* < .001). Mechanical ventilation (64.5%) and venoarterial extracorporeal membrane oxygenation (VA-ECMO) (87.4%) were particularly common in Status 0. In contrast, ventricular assist devices (VADs) were more often used in Status 1–3 (6.6% vs. 12.8%, *P* = .006), while intra-aortic balloon pump (IABP) use was similar (1.4% vs. 0.2%, *P* = .134). Renal replacement therapy (RRT) was also more common in Status 0 than in Status 1–3 (43.2% vs. 4.3%, *P* < .001). After heart transplantation, Status 0 recipients continued to have higher VA-ECMO (24.4% vs. 4.8%), RRT (41.5% vs. 11.1%), and use of prolonged mechanical ventilation for >24 h (72.5% vs. 31.7%) use (all *P* < .001) (Supplementary Figure S6). To assess whether differences in post-transplant infection risk by pre-transplant urgency status persisted after adjustment for pre- and post-transplant support, we performed multivariable Cox regression analyses including mechanical and organ support variables (Supplementary Table S4). In univariable analyses, Status 0 was associated with a higher risk of post-transplant infection at 1 and 6 months. However, this association was no longer significant after adjustment for MCS- and critical care-related factors, including VA-ECMO, VADs, RRT, and mechanical ventilation. Prolonged mechanical ventilation (>24 h) after heart transplantation was the strongest independent predictor of infection at both 1 and 6 months post-transplant. Consistent with this finding, an additional analysis restricted to Status 0 recipients showed that pre-transplant ventilator support was also associated with 30-day post-transplant infection. In the final multivariable Cox model, pre-transplant ventilator support, baseline haemoglobin level, recipient age at transplantation, and baseline white blood cell count were identified as predictors of 30-day post-transplant infection. An internally validated prediction model based on pre-transplant variables further demonstrated acceptable discrimination and stratified Status 0 recipients into low- and high-risk groups with distinct observed 30-day infection rates (Supplementary Figure S7). Furthermore, Status 0 recipients who required prolonged mechanical ventilation after heart transplantation had a significantly higher risk of infection than both Status 0 recipients with early ventilator weaning and Status 1–3 recipients with prolonged ventilation, and this trend persisted through 6 months (Supplementary Figure S8).

### Pathogens and infection sites within the first year after transplantation

Among recipients who developed post-transplant infections within the first year, bacteria were the most commonly identified pathogens, followed by fungi and viruses. At 1 month after transplantation, bacterial infections (25.6% vs. 7.3%, *P* < .001) and fungal infections (12.6% vs. 3.2%, *P* < .001) were significantly more frequent in Status 0 recipients than in Status 1–3 recipients. Bacteremia (6.7% vs. 0.8%, *P* < .001) and fungemia (2.6% vs. 0.2%, *P* = .003) were also more common in Status 0 recipients. At 6 months after transplantation, bacterial infections (16.4% vs. 5.0%, *P* < .001) and bacteremia (3.5% vs. 0.2%, *P* = .001) remained significantly more frequent in Status 0 recipients. The respiratory tract was the most common site of infection, and respiratory tract infections were more frequent in Status 0 recipients than in Status 1–3 recipients within 12 months after transplantation (*Table 2*).

**Table 2:** Infection sites and pathogens during the first year after transplantation

One month after transplantation	Status 0 (*n* = 270)	Status 1–3 (*n* = 507)	*P*-value
Pathogens−no. (%)			
Bacterial infection	69 (25.6)	37 (7.3)	<.001
Bacteremia	18 (6.7)	4 (0.8)	<.001
Viral infection	8 (3.0)	9 (1.8)	.308
Fungal infection	34 (12.6)	16 (3.2)	<.001
Fungemia	7 (2.6)	1 (0.2)	.003
Unknown/others	1 (0.4)	6 (1.2)	.432
Total infection	112 (41.5)	68 (13.4)	<.001
Sites−no. (%)			
Wound	10 (3.7)	7 (1.4)	.042
Allograft	0 (0.0)	1 (0.2)	1.000
Respiratory tract	49 (18.1)	30 (5.9)	<.001
Gastrointestinal tract	12 (4.4)	6 (1.2)	.010
Urinary tract	5 (1.9)	2 (0.4)	0.053
Skin	2 (0.7)	5 (1.0)	1.000
Musculoskeletal	0 (0.0)	0 (0.0)	n/a

Six months after transplantation	Status 0 (*n* = 226)	Status 1–3 (*n* = 458)	*P*-value
Pathogens−no. (%)			
Bacterial infection	37 (16.4)	23 (5.0)	<.001
Bacteremia	8 (3.5)	1 (0.2)	.001
Viral infection	8 (3.5)	22 (4.8)	.553
Fungal infection	15 (6.6)	21 (4.6)	.277
Fungemia	0 (0.0)	2 (0.4)	1.000
Unknown/others	3 (1.3)	10 (2.2)	.561
Total infection	63 (27.9)	76 (16.6)	.001
Sites−no. (%)			
Wound	6 (2.7)	4 (0.9)	.090
Allograft	3 (1.3)	0 (0.0)	.036
Respiratory tract	23 (10.2)	21 (4.6)	.007
Gastrointestinal tract	8 (3.5)	6 (1.3)	.081
Urinary tract	6 (2.7)	7 (1.5)	.373
Skin	2 (0.9)	6 (1.3)	1.000
Musculoskeletal	1 (0.4)	3 (0.7)	1.000

12 months after transplantation	Status 0 (*n* = 199)	Status 1–3 (*n* = 427)	*P*-value
Pathogens−no. (%)			
Bacterial infection	15 (7.5)	18 (4.2)	.087
Bacteremia	3 (1.5)	2 (0.5)	.333
Viral infection	8 (4.0)	10 (2.3)	.304
Fungal infection	5 (2.5)	7 (1.6)	.533
Fungemia	1 (0.5)	0 (0.0)	1.000
Unknown/others	8 (4.0)	9 (2.1)	.190
Total infection	36 (18.1)	44 (10.3)	.010
Sites−no. (%)			
Wound	0 (0.0)	0 (0.0)	n/a
Allograft	0 (0.0)	0 (0.0)	n/a
Respiratory tract	16 (8.0)	12 (2.8)	.006
Gastrointestinal tract	1 (0.5)	6 (1.4)	.432
Urinary tract	4 (2.0)	4 (0.9)	.267
Skin	3 (1.5)	7 (1.6)	1.000
Musculoskeletal	2 (1.0)	3 (0.7)	1.000

Abbreviation: n/a, not applicable

### Immunosuppressant exposure and risk of post-transplant infection

At hospital discharge after heart transplantation, tacrolimus trough levels were higher in Status 0 recipients than in Status 1–3 recipients. However, from 1 month to 1 year after transplantation, tacrolimus levels were consistently lower in Status 0. For mycophenolate mofetil (MMF), the prescribed dose at discharge was similar between groups, whereas from 1 month to 1 year after transplantation, MMF doses were lower in Status 0 recipients than in Status 1–3 recipients. In contrast, steroid dosing showed an opposite pattern, with Status 0 recipients receiving lower steroid doses at discharge but higher steroid doses from 1 month to 1 year after transplantation compared with Status 1–3 recipients (*Table 3*). Among Status 0 recipients, failure to taper corticosteroids to ≤5 mg/day or discontinue corticosteroids within 6 months after transplantation was associated with a significantly higher risk of post-transplant infection at 6 months compared with successful tapering to ≤5 mg/day or discontinuation within 6 months. In contrast, among Status 1–3 recipients, the risk of post-transplant infection at 6 months did not differ significantly according to whether corticosteroids were tapered or discontinued within 6 months (Supplementary Figure S9A). The association between steroid tapering and lower infection risk was significant in Status 0 recipients but not in Status 1–3 during the early post-transplant period, with a significant interaction by urgency status (*P* for interaction = .008), suggesting a differential association across urgency groups. Although early steroid tapering or discontinuation was associated with numerically higher risk of rejection within 6 months compared with maintenance therapy, the difference was not statistically significant. This pattern was observed consistently in both Status 0 and Status 1–3 recipients (Supplementary Figure S9B).

**Table 3:** Comparison of prescribed immunosuppressant doses or serum drug levels according to urgency status

Immunosuppressant dosages	Status 0 (*n* = 287)	Status 1 (*n* = 451)	Status 2,3 (*n* = 63)	*P*-value
At 1 month after transplantation				
Tacrolimus (ng/ml)	8.3 ± 3.0	8.9 ± 3.1	10.4 ± 4.0	<.001
MMF (mg)	1176.3 ± 605.1	1319.5 ± 544.0	1259.3 ± 597.3	.012
Steroid (mg)	16.9 ± 8.3	16.9 ± 8.7	14.1 ± 6.6	.041
Everolimus (ng/ml)	3.9 ± 2.1	3.4 ± 1.6	3.3 ± 1.4	.726
At 6 months after transplantation				
Tacrolimus (ng/ml)	7.1 ± 3.2	7.7 ± 3.2	8.2 ± 3.8	.022
MMF (mg)	1000.0 ± 573.1	1146.4 ± 589.1	1096.9 ± 580.4	.018
Steroid (mg)	8.2 ± 10.1	6.0 ± 3.6	6.2 ± 2.4	.001
Everolimus (ng/ml)	3.7 ± 1.4	4.1 ± 2.0	3.8 ± 2.1	.288
At 1 year after transplantation				
Tacrolimus (ng/ml)	6.5 ± 2.8	7.1 ± 3.3	6.9 ± 2.5	.115
MMF (mg)	940.7 ± 538.5	1077.2 ± 595.7	1064.4 ± 599.2	.037
Steroid (mg)	6.1 ± 7.0	4.1 ± 1.7	4.7 ± 3.1	<.001
Everolimus (ng/ml)	4.3 ± 1.7	4.3 ± 1.9	3.4 ± 1.2	.089

Abbreviation: MMF, mycophenolate mofetil

Tacrolimus and everolimus are expressed as a serum trough concentration

## Discussion

In this nationwide cohort study, we demonstrated that post-transplant outcomes differed significantly according to pre-transplant urgency status within the KONOS heart allocation system. Although acute allograft rejection, infection, and CAV are well-established complications after heart transplantation, early post-transplant infection emerged as the most important determinant of mortality in our cohort. In particular, recipients classified as Status 0 had a significantly higher risk of infection at 1 and 6 months after transplantation, and these early post-transplant infections were strongly associated with increased mortality and poorer long-term outcomes.

The KONOS heart allocation system was revised in 2017 to better reflect clinical severity and waitlist mortality risk, particularly among high-risk candidates requiring temporary MCS or intensive care. Because our study period included this policy revision, we performed a sensitivity analysis using the pre-revision KONOS urgency criteria. Even under the pre-revision criteria, Status 0 recipients had higher post-transplant mortality and infection risk than Status 1–3 recipients, consistent with the main analysis based on the revised criteria. These findings indicate that the adverse outcomes observed in Status 0 recipients were not primarily attributable to the 2017 classification change. Instead, they support the interpretation that Status 0 reflects a clinically meaningful high-risk phenotype with greater baseline severity and increased susceptibility to early post-transplant infection and mortality. This early vulnerability among Status 0 recipients may be explained, at least in part, by their substantially higher baseline clinical severity. In our cohort, recipients assigned to Status 0 represented a distinctly high-risk population characterized by a greater burden of comorbidities and advanced heart failure at the time of transplantation. Compared with Status 1–3 recipients, Status 0 patients had a higher prevalence of diabetes mellitus and chronic kidney disease, lower left ventricular ejection fraction, and markedly elevated natriuretic peptide levels. In addition, they were substantially more likely to require invasive therapies, including mechanical ventilation, temporary MCS, and RRT, both before and after transplantation. While these clinical features appropriately conferred the highest priority under the KONOS allocation system and facilitated earlier transplantation, they simultaneously increased cumulative exposure to invasive interventions, thereby amplifying vulnerability to post-transplant infection and mortality. These findings are consistent with prior reports linking pre-transplant mechanical ventilation and temporary MCS to adverse post-transplant outcomes,^17–20^ as well as studies identifying advanced age, diabetes mellitus, and invasive respiratory or circulatory support as major risk factors for post-transplant infections.^21–27^ In our univariable Cox regression analyses, Status 0 was associated with a significantly higher risk of early post-transplant infection. However, this association was no longer significant after adjustment for MCS- and critical care-related factors, suggesting that the excess infection risk in Status 0 recipients was largely explained by their greater clinical severity and dependence on intensive supportive therapies rather than by urgency status itself. Interestingly, although ECMO has frequently been implicated as a contributor to infection risk in previous studies,^24–27^ VA-ECMO alone was not independently associated with early post-transplant infection in our cohort. Instead, more than half (55.7%) of Status 0 recipients required concomitant VA-ECMO and mechanical ventilation during the waiting period, suggesting that the cumulative burden of invasive support, rather than ECMO itself, may underlie the observed excess risk. This distinction is clinically relevant, as it underscores the importance of evaluating the combined and prolonged effects of invasive therapies when assessing post-transplant infectious risk in high-urgency recipients. The extended Cox model incorporating a time-dependent interaction term showed that the relative effect of Status 0 on post-transplant infection decreased over time, as indicated by the significant urgency status × log(time) interaction. This finding suggests that the infection vulnerability associated with Status 0 was concentrated primarily in the early post-transplant period rather than persisting uniformly throughout follow-up. However, the observed attenuation of HRs over time should be interpreted cautiously, because it may also partly reflect survivor selection, whereby patients at the highest risk experience infection or death early, leaving a relatively lower-risk population at later time points. Thus, Status 0 appears to represent a clinically meaningful high-risk phenotype with early post-transplant infection vulnerability, partly mediated by MCS dependence, critical illness, and potential survivor selection during follow-up.

Among the factors evaluated in this study, prolonged mechanical ventilation was the strongest independent predictor of post-transplant infection within the first year, particularly among Status 0 recipients. Pre-transplant ventilator support also independently predicted 30-day post-transplant infection in this group, suggesting that respiratory vulnerability may begin during the waiting period and persist after transplantation. Accordingly, prolonged post-transplant mechanical ventilation may represent the continuation of pre-existing critical illness rather than an isolated post-operative complication, providing a mechanistic explanation for the higher infection and mortality burden in Status 0 recipients. This interpretation is supported by the predominance of bacterial and respiratory tract infections in our cohort. Importantly, even among Status 0 recipients, early liberation from ventilatory support within 24 h after transplantation was associated with significantly reduced risks of infection and mortality, suggesting that early weaning from mechanical ventilation may represent an important therapeutic target. Future studies are warranted to identify factors associated with early weaning and to determine whether strategies such as lung-protective ventilation, early weaning protocols, rehabilitation, and prophylactic antibiotic approaches can effectively reduce post-transplant infections and infection-related mortality in this high-risk population.

In contrast to infection-related outcomes, there were no significant differences among urgency status groups in the risk of acute allograft rejection, CAV, or related mortality during the follow-up period. This finding may reflect recent advances in immunosuppressive therapy, individualized immunosuppressive strategies, and routine rejection surveillance, all of which have contributed to reducing adverse outcomes related to rejection and CAV. However, although the overall incidence of rejection and CAV was similar between the two groups during follow-up, the proportion of patients requiring re-transplantation due to rejection, graft failure, or CAV was higher among Status 0 recipients (Supplementary Table S5). This suggests that post-transplant complications in Status 0 recipients were more likely to progress beyond isolated clinical events to clinically significant graft dysfunction severe enough to require re-transplantation. These findings indicate that, in addition to increased vulnerability to infection during the early post-transplant period, Status 0 recipients remain at sustained long-term risk of re-transplantation related to rejection or CAV. Furthermore, re-transplantation itself may increase susceptibility to infection through additional surgical stress, intensified immunosuppression, and prolonged intensive care exposure. Taken together, these findings highlight the challenge of maintaining an optimal balance of immunosuppressive therapy in high-risk transplant recipients. Excessive immunosuppression is a major contributor to post-transplant infections,^28^ whereas insufficient immunosuppression can lead to allograft rejection. In Korea, triple therapy consisting of tacrolimus, MMF, and steroids remains the standard maintenance immunosuppressive regimen during the early post-transplant period. Over time, however, maintenance immunosuppression is gradually modified, typically through steroid tapering or withdrawal and the introduction of everolimus.^29^ In the present study, we observed distinct temporal differences in immunosuppressive regimens during post-transplant follow-up between Status 0 and Status 1–3 recipients. Compared with Status 1–3 recipients, Status 0 recipients had higher tacrolimus trough levels and received relatively lower steroid doses at hospital discharge after transplantation. However, from 1 month to 1 year after transplantation, tacrolimus trough levels and MMF doses were maintained at relatively lower levels, whereas steroid doses remained relatively higher. These differences likely reflect the unique clinical characteristics of Status 0 recipients, who had a greater comorbidity burden at transplantation, including ischaemic heart disease, diabetes mellitus, and chronic kidney disease, as well as greater disease severity reflected by the need for RRT during the waiting period or after transplantation. These factors may directly influence immunosuppressive strategy decisions in real-world clinical practice. During the early post-transplant period, high tacrolimus trough levels may have been intentionally maintained in Status 0 recipients to prevent acute rejection. In addition, greater early tacrolimus exposure may have allowed the use of relatively lower initial steroid doses. During subsequent follow-up, however, tacrolimus use may have been limited by concerns regarding deterioration in renal function. As a result, maintenance steroid therapy may have been prolonged, or some patients may have shifted towards corticosteroid-dependent immunosuppressive strategies for rejection prevention. Nevertheless, our findings do not allow us to determine whether these temporal changes in immunosuppressive regimens or the relatively higher steroid exposure observed at specific time points in Status 0 recipients were directly associated with infection risk or infection-related mortality. Therefore, these findings should be interpreted not as evidence that higher steroid exposure directly caused poor outcome, but rather as a reflection of the underlying clinical complexity of Status 0 recipients. In real-world practice, immunosuppressive management is not simply the application of a standardized protocol, but a complex therapeutic decision-making process that requires comprehensive consideration of patient comorbidities and the post-transplant clinical course. These findings suggest that achieving an optimal balance of immunosuppression—avoiding both excessive immunosuppression leading to infection and insufficient immunosuppression leading to rejection—may be more challenging in Status 0 recipients than in Status 1–3 recipients, particularly during the early post-transplant period.

Several limitations of this study should be acknowledged. First, as a retrospective observational analysis of registry data, the study is subject to potential selection bias and residual confounding, particularly given the inherent clinical severity of Status 0 recipients. Second, the study population consisted primarily of Asian patients, which may limit generalizability to other regions. Third, device availability and usage patterns differ across countries. In Korea, VA-ECMO, IABP, and durable VADs are the predominant modalities, and the relatively small number of patients receiving certain devices limited subgroup analyses. Lastly, detailed microbiological data were unavailable, precluding pathogen-specific analyses. Future studies incorporating granular microbiological profiling and prospective data collection are warranted.

In conclusion, allocation-driven high-urgency heart transplantation was associated with a distinct pattern of outcome trade-offs, in which early post-transplant infection emerged as the principal determinant of mortality. This vulnerability appeared to be largely related to prolonged mechanical ventilation and the underlying clinical complexity of Status 0 recipients, including challenges in balancing immunosuppressive therapy. These findings emphasize the need for enhanced peri- and post-transplant management strategies tailored to high-urgency recipients to mitigate infection risk and improve long-term outcomes.

## Supplementary Material

xvag167_Supplementary_Data

## Declarations

### Data Availability

No data were generated or analysed for this manuscript.

### Pre-registered Clinical Trial Number

None supplied.

## Apendix

**Members of the Korean Organ Transplant Registry (KOTRY) Investigators:** In Cheol Kim, Division of Cardiology, Keimyung University Dongsan Medical Center, Daegu, South Korea. Jaewon Oh, Department of Internal Medicine, Yonsei University College of Medicine, Seoul, South Korea. Jin Joo Park, Division of Cardiology, Department of Internal Medicine, Seoul National University Bundang Hospital, Seongnam, Gyeonggi, South Korea. Min Ho Ju, Department of Thoracic and Cardiovascular Surgery, Pusan National University Yangsan Hospital, Pusan, South Korea. Sung Ho Jung, Department of Thoracic and Cardiovascular Surgery, Asan Medical Center, University of Ulsan College of Medicine, Seoul, South Korea. Jung Ae Hong, Department of Thoracic and Cardiovascular Surgery, Asan Medical Center, University of Ulsan College of Medicine, Seoul, South Korea. Jeehoon Kang, Department of Internal Medicine, Seoul National University Hospital, Seoul, South Korea. Jeongsu Kim, Division of Cardiology, Pusan National University Yangsan Hospital, Pusan, South Korea. Junho Hyun, Department of Internal Medicine, Asan Medical Center, University of Ulsan College of Medicine, Seoul, South Korea. Mi Hee Lim, Department of Thoracic and Cardiovascular Surgery, Pusan National University Yangsan Hospital, Pusan, South Korea. Minjae Yoon, Division of Cardiology, Department of Internal Medicine, Seoul National University Bundang Hospital, Seongnam, Gyeonggi, South Korea. Nam Hee Park, Department of Thoracic and Cardiovascular Surgery, Keimyung University Dongsan Medical Center, Daegu, South Korea. Sun Hwa Lee, Division of Cardiology, Keimyung University Dongsan Medical Center, Daegu, South Korea.
