# Supplementary material for: High-urgency heart transplantation and outcome trade-offs: early post-transplant infection and mortality
Source: ESC Heart Fail. 2026 Jun 10;13(3):xvag167. doi: 10.1093/eschf/xvag167 (PMC13298874; doi:10.1093/eschf/xvag167)
Supplement: xvag167_Supplementary_Data [file xvag167_supplementary_data.docx]

**Supplemental Materials**

**High-Urgency Heart Transplantation and Outcome Trade-offs: Early Post-Transplant Infection and Mortality**

**Lee KS et al.**

**Table Contents**

#1. Table S1.

2017 Korean Network for Organ Sharing (KONOS) donor heart allocation system

#2. Table S2.

HRs (95% confidence interval) for all-cause mortality based on infection, rejection, and cardiac allograft vasculopathy events during the follow-up

#3. Table S3.

Time-dependent Cox regression hazard ratios for 1-year post-transplant infection

#4. Table S4.

Univariable and multivariable Cox proportional hazards models for 1- and 6-month post-transplant infection

#5. Table S5.

Frequency and causes of re-transplantation observed during the follow-up period in the Korean Organ Transplant Registry (KOTRY), 2014–2021**Figure Contents**

#1. Figure S1

Study flow

#2. Figure S2

Post-transplant rejection risk by Urgency Status

#3. Figure S3

Sensitivity analysis of clinical outcomes according to Urgency Status using KONOS criteria before the 2017 revision

#4. Figure S4

Temporal impact of infection, rejection, and cardiac allograft vasculopathy on all-cause mortality after heart transplantation

#5. Figure S5

Mediation analysis assessing the role of post-transplant infection in the relationship between Urgency Status and all-cause mortality

#6. Figure S6

Comparison of the use of pre- and post-transplant supportive interventions and mechanical circulatory support (MCS) devices by Urgency Status

#7. Figure S7

Pre-transplant risk factors and risk stratification model for 30-day post-transplant infection in Status 0 recipients.

#8. Figure S8

Impact of post-transplant ventilation duration on early post-transplant infection risk by Urgency Status

#9. Figure S9

Impact of steroid tapering on early post-transplant infection and rejection risk by Urgency Status

**Table S1. 2017 Korean Network for Organ Sharing (KONOS) donor heart allocation system**

| Status 0 | At least one of the following therapy  (Re-registration in 8 days) |
| --- | --- |
|  | (1) VT/VF needs VAD |
|  | (2) VT/VF needs IABP |
|  | (3) VA-ECMO |
|  | (4) VAD with serious complication (thromboembolism, device infection, mechanical failure, recurrent ventricular arrhythmia) who needs admission at the ICU |
|  | (5) Requiring continuous mechanical ventilation due to heart failure |
|  | (6) External VAD (RVAD, LVAD, Bi-VAD) |
| Status 1 | Inpatient, at least one of the following (Re-registration in 8 days) |
|  | 1) Artificial heart |
|  | 2) VAD (no need of admission) |
|  | 3) IABP |
|  | 4) Continuous infusion of IV inotropes for more than 4 weeks |
|  | 5) Continuous infusion of a single high-dose IV inotrope or multiple moderate dose IV inotropes for 1 week |
|  | 6) VT/VF for more than 3 times/24 hours (despite the antiarrhythmics or the previous anti-arrhythmic procedure) or more than 3 ICD shock events during re-registration period |
| Status 2 | (Re-registration in 1 month) |
|  | Continuous infusion of IV inotropes but not fulfilling criteria for Status 1 |
|  | VT/VF despite the antiarrhythmics or the previous anti-arrhythmic procedure, ICD shock |
|  | (but not fulfilling criteria for Status 1) |
| Status 3 | A patient who does not meet the criteria for Status 0, 1 or 2 |
| Status 7 | Deferred for heart transplantation listing |

Abbreviations: VT/VF, ventricular tachycardia/ventricular fibrillation; VAD, ventricular assist device; IABP, intra-aortic balloon pump; VA-ECMO, veno-arterial extra-corporeal membrane oxygenation; ICU, intensive care unit; IV, intravenous; ICD, implantable cardioverter-defibrillator; LVAD, left ventricular assist device; RVAD, right ventricular assist device; Bi-VAD, Biventricular assist device.

**Table S2. HRs (95% confidence interval) for all-cause mortality based on infection, rejection, and cardiac allograft vasculopathy events during the follow-up**

| Post-transplant  time points | Infection (-)  no. (%) | Infection (+)  no. (%) | HR* (95% CI) | *P*-value |
| --- | --- | --- | --- | --- |
| At 1month | 52/606 (8.6) | 55/171 (32.2) | 2.81 (1.63−4.86) | <0.001 |
| At 6months | 37/554 (6.7) | 30/130 (23.1) | 3.44 (1.98−5.98) | <0.001 |
| At 1-year | 32/548 (5.8) | 19/78 (24.4) | 2.44 (1.37−4.35) | 0.002 |
| At 2-year | 23/446 (5.2) | 9/68 (13.2) | 1.50 (0.81−2.77) | 0.200 |
| At 3-year | 14/379 (3.7) | 6/34 (17.6) | 1.71 (0.60−4.83) | 0.314 |
| At 4-year | 10/312 (3.2) | 2/14 (14.3) | 1.35 (0.19−10.52) | 0.743 |
| At 5-year | 8/216 (3.7) | 2/11 (18.2) | 2.56 (0.60−11.16) | 0.205 |
| Post-transplant  time points | Rejection (-)  no. (%) | Rejection (+)  no. (%) | HR* (95% CI) | *P*-value |
| At 1month | 95/686 (13.8) | 12/91 (13.2) | 1.07 (0.50-2.29) | 0.856 |
| At 6months | 53/542 (9.8) | 14/160 (8.8) | 1.90 (0.93-3.85) | 0.076 |
| At 1-year | 44/541 (8.1) | 7/85 (8.2) | 1.41 (0.58-3.41) | 0.450 |
| At 2-year | 30/504 (6.0) | 2/10 (20.0) | 2.41 (0.28-20.78) | 0.423 |
| At 3-year | 19/408 (4.7) | 1/5 (20.0) | - | - |
| At 4-year | 10/323 (3.1) | 2/3 (66.7) | - | - |
| At 5-year | 9/224 (4.0) | 1/3 (33.3) | - | - |
| Post-transplant  time points | Cardiac allograft vasculopathy (-)  no. (%) | Cardiac allograft vasculopathy (+)  no. (%) | HR* (95% CI) | *P*-value |
| At 1month | 107/777 (13.8) | 0/0 (0.0) | - | - |
| At 6months | 67/684 (9.8) | 0/0 (0.0) | - | - |
| At 1-year | 50/592 (8.4) | 1/34 (2.9) | 0.32 (0.04-2.33) | 0.260 |
| At 2-year | 32/483 (6.6) | 0/31 (0.0) | - | - |
| At 3-year | 19/363 (5.2) | 1/50 (2.0) | 0.34 (0.05-2.61) | 0.301 |
| At 4-year | 11/281 (3.9) | 1/45 (2.2) | 0.45 (0.06-3.62) | 0.453 |
| At 5-year | 8/192 (4.2) | 2/35 (5.7) | 1.17 (0.23-5.97) | 0.849 |

Abbreviations: HR, hazard ratio; CI, confidence interval

*Covariates for adjustment: Age, Sex, BMI, causes of transplantation (ischemic heart disease, cardiomyopathy), DM, hypertension, chronic kidney disease, operation time, cold ischemic time, panel-reactive antibody, baseline natriuretic peptide levels, and urgency status

**Table S3. Time-dependent Cox regression hazard ratios for 1-year post-transplant infection**

| Variable | HR | 95% CI lower | 95% CI  upper | *P* -value |
| --- | --- | --- | --- | --- |
| Chronic kidney disease | 0.15 | 0.07 | 0.35 | <0.005 |
| Panel-reactive antibody≥50% | 0.19 | 0.04 | 0.87 | 0.030 |
| HTx for cardiomyopathy (logT) | 0.45 | 0.36 | 0.58 | <0.005 |
| Desensitization (logT) | 0.51 | 0.33 | 0.78 | <0.005 |
| Assist devices (logT) | 0.52 | 0.39 | 0.68 | <0.005 |
| Comorbidities (logT) | 0.59 | 0.47 | 0.74 | <0.005 |
| Urgency Status group (logT) | 0.62 | 0.48 | 0.80 | <0.005 |
| HTx for IHD (logT) | 0.66 | 0.50 | 0.86 | <0.005 |
| Renal replacement therapy (logT) | 0.66 | 0.52 | 0.85 | <0.005 |
| Hypertension | 0.73 | 0.35 | 1.53 | 0.400 |
| Diabetes mellitus (logT) | 0.85 | 0.70 | 1.04 | 0.110 |
| PHM match category | 0.95 | 0.71 | 1.27 | 0.710 |
| Recipient age at TPL (logT) | 0.97 | 0.96 | 0.98 | <0.005 |
| Post ventilator duration (logT) | 0.98 | 0.97 | 0.99 | <0.005 |
| Operation time (logT) | 1.00 | 1.00 | 1.00 | <0.005 |
| Cold ischemic time (logT) | 1.00 | 0.99 | 1.00 | <0.005 |
| Time from listing to TPL (days) | 1.00 | 1.00 | 1.00 | 0.850 |
| Warm ischemic time (logT) | 1.00 | 0.99 | 1.00 | 0.040 |
| Time from listing to TPL (logT) | 1.00 | 1.00 | 1.00 | 0.840 |
| Warm ischemic time (min) | 1.01 | 1.00 | 1.02 | 0.1400 |
| Operation time (min) | 1.01 | 1.01 | 1.01 | <0.005 |
| Cold ischemic time (min) | 1.01 | 1.01 | 1.02 | <0.005 |
| Post ventilator duration (day) | 1.07 | 1.03 | 1.10 | <0.005 |
| Steroid tapered off | 1.07 | 0.79 | 1.44 | 0.67 |
| Hypertension (logT) | 1.13 | 0.94 | 1.37 | 0.200 |
| Recipient age at TPL (year) | 1.13 | 1.09 | 1.16 | <0.005 |
| Panel-reactive antibody≥50% (logT) | 1.39 | 0.94 | 2.06 | 0.100 |
| Chronic kidney disease (logT) | 1.68 | 1.34 | 2.10 | <0.005 |
| Diabetes mellitus | 1.73 | 0.85 | 3.48 | 0.130 |
| Urgency Status group | 2.24 | 0.80 | 6.29 | 0.130 |
| Renal replacement therapy | 4.32 | 1.85 | 10.06 | <0.005 |
| Assist devices | 5.24 | 1.79 | 15.33 | <0.005 |
| HTx for IHD | 7.05 | 2.28 | 21.76 | <0.005 |
| Desensitization | 12.38 | 3.09 | 49.57 | <0.005 |
| Comorbidities | 14.34 | 5.16 | 39.86 | <0.005 |
| HTx for cardiomyopathy | 18.82 | 6.72 | 52.72 | <0.005 |

Abbreviations: HR, hazard ratio; CI, confidence interval; HTx, heart transplantation; IHD, ischemic heart disease PHM, predicted heart mass; TPL, transplantation

Log-transformed variables (indicated as logT) represent time-updated covariates that vary across follow-up, allowing more accurate modeling of dynamic clinical events. A time-dependent Cox model was used to appropriately account for such non-proportional and time-varying effects, improving the validity of hazard estimation in the post-transplant population.

**Table S4. Univariable and multivariable Cox proportional hazards models for 1- and 6-month post-transplant infection**

| Model for 1-month infection | Variable | HR (95% CI) | *P*-value |
| --- | --- | --- | --- |
| Univariable | Urgency status (Status 0 vs. Status 1ￚ3) | 3.57 (2.63−4.76) | < 0.005 |
| ¶Multivariable | Urgency status (Status 0 vs. Status 1ￚ3) | 1.15 (0.58−2.27) | 0.680 |
|  | Mechanical ventilation | 1.40 (0.89−2.22) | 0.150 |
|  | VA-ECMO | 1.13 (0.92−1.39) | 0.230 |
|  | Ventricular assist device | 1.64 (1.02 −2.65) | 0.040 |
|  | IABP | 2.07 (0.49−8.66) | 0.320 |
|  | Renal replacement therapy (RRT) | 1.39 (0.93−2.09) | 0.110 |
|  | Post-transplant VA-ECMO | 1.34 (0.93 – 1.93) | 0.110 |
|  | Post-transplant RRT | 1.29 (0.87 – 1.91) | 0.210 |
|  | Prolonged ventilation after transplantation | 2.30 (1.56 – 3.38) | < 0.005 |
| Model for 6-months infection | Variable | HR (95% CI) | P-value |
| Univariable | Urgency status (Status 0 vs. Status 1ￚ3) | 2.50 (1.96−3.13) | < 0.005 |
| ¶Multivariable | Urgency status (Status 0 vs. Status 1ￚ3) | 1.03 (0.57 – 1.85) | 0.920 |
|  | Mechanical ventilation | 1.35 (0.91 – 2.01) | 0.140 |
|  | VA-ECMO | 1.09 (0.91 – 1.30) | 0.350 |
|  | Ventricular assist device | 1.15 (0.76 – 1.74) | 0.520 |
|  | Renal replacement therapy (RRT) | 1.40 (0.99 – 1.99) | 0.006 |
|  | IABP | 1.51 (0.36 – 6.29) | 0.570 |
|  | Post-transplant VA-ECMO | 1.10 (0.79 – 1.53) | 0.580 |
|  | Post-transplant RRT | 1.38 (0.99 – 1.92) | 0.060 |
|  | Prolonged ventilation after transplantation | 2.01 (1.50 – 2.71) | < 0.005 |

Abbreviations: HR, hazard ratio; CI, confidence interval; VA-ECMO, veno-arterial extracorporeal membrane oxygenation; IABP, intra-aortic balloon pump

¶Multivariable models were adjusted for urgency status and pre-transplant support variables (mechanical ventilation, VA-ECMO, VAD, RRT, and IABP) and post-transplant variables (VA-ECMO, RRT, and duration of mechanical ventilation).

**Table S5. Frequency and causes of re-transplantation observed during the follow-up period in the Korean Organ Transplant Registry (KOTRY), 2014–2021**

| Causes for re-transplantation | Status 0  (n=287) | Status 1ￚ3  (n=514) | *P*-value |
| --- | --- | --- | --- |
| Acute rejection−no. (%) | 4 (1.4) | 3 (0.6) | - |
| Chronic rejection−no. (%) | 5 (1.7) | 3 (0.6) | - |
| Primary graft failure−no. (%) | 4 (1.4) | 4 (0.8) | - |
| Cardiac allograft vasculopathy−no. (%) | 4 (1.4) | 5 (1.0) | - |
| Others−no. (%) | 4 (1.4) | 0 | - |
| Cardiac arrest and arrhythmia | 2 (0.7) | 0 | - |
| Pericarditis | 1 (0.4) | 0 | - |
| Ischemic heart disease | 1 (0.4) | 0 | - |
| Total | 21 (7.3) | 15 (2.9) | 0.007 |

**Figure S1. Study flow**


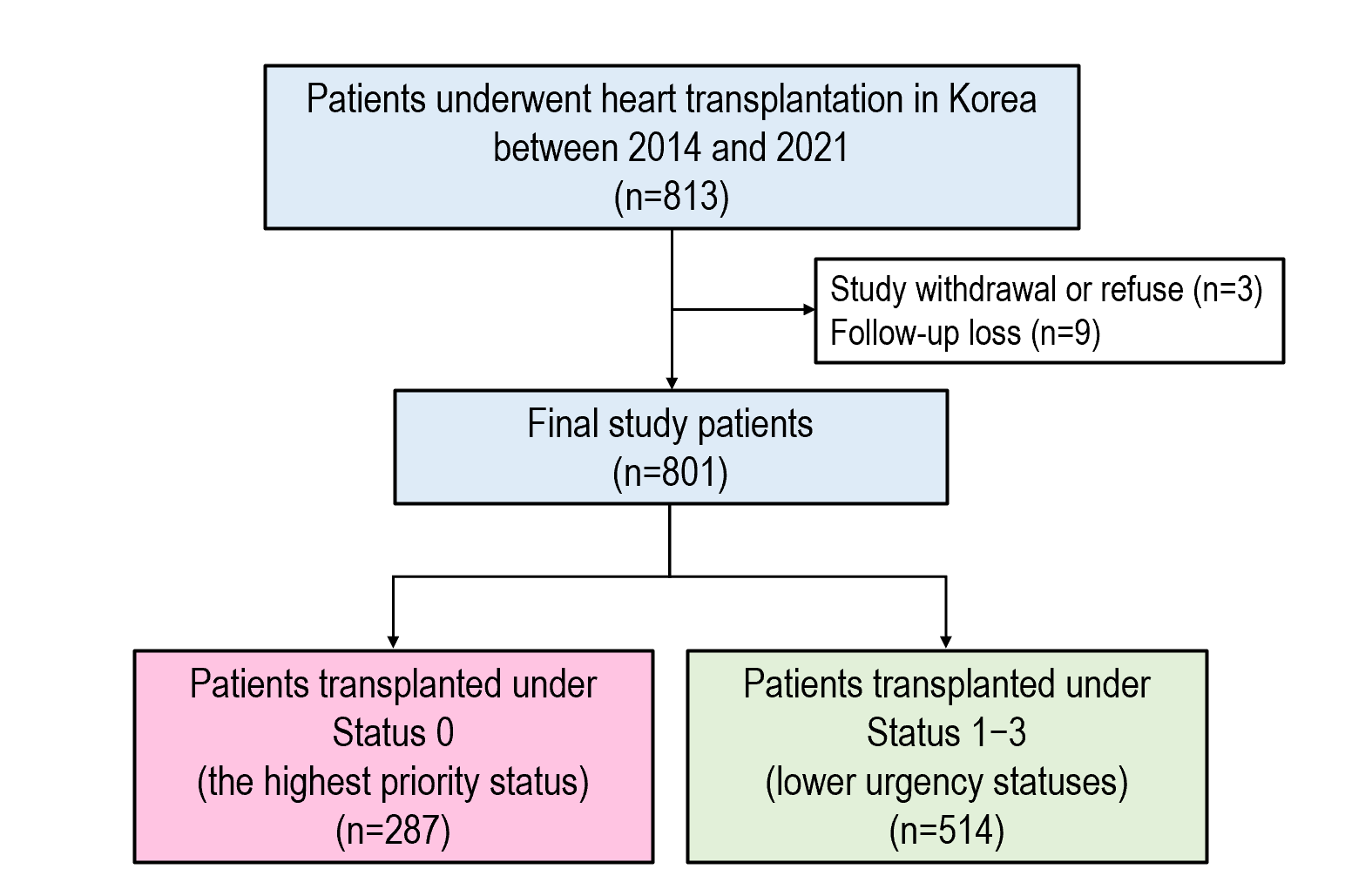


This flowchart illustrates the selection process for the final study population. Among 813 patients who underwent heart transplantation in Korea between 2014 and 2021, 3 patients were excluded due to study withdrawal or refusal, and 9 patients were lost to follow-up. The final analysis included 801 patients, who were then stratified into two groups based on Urgency Status at the time of transplant: Status 0 (n = 287), representing the highest priority patients, and Status 1ￚ3 (n = 514), representing patients with lower urgency.

**Figure S2. Post-transplant rejection risk by urgency status**


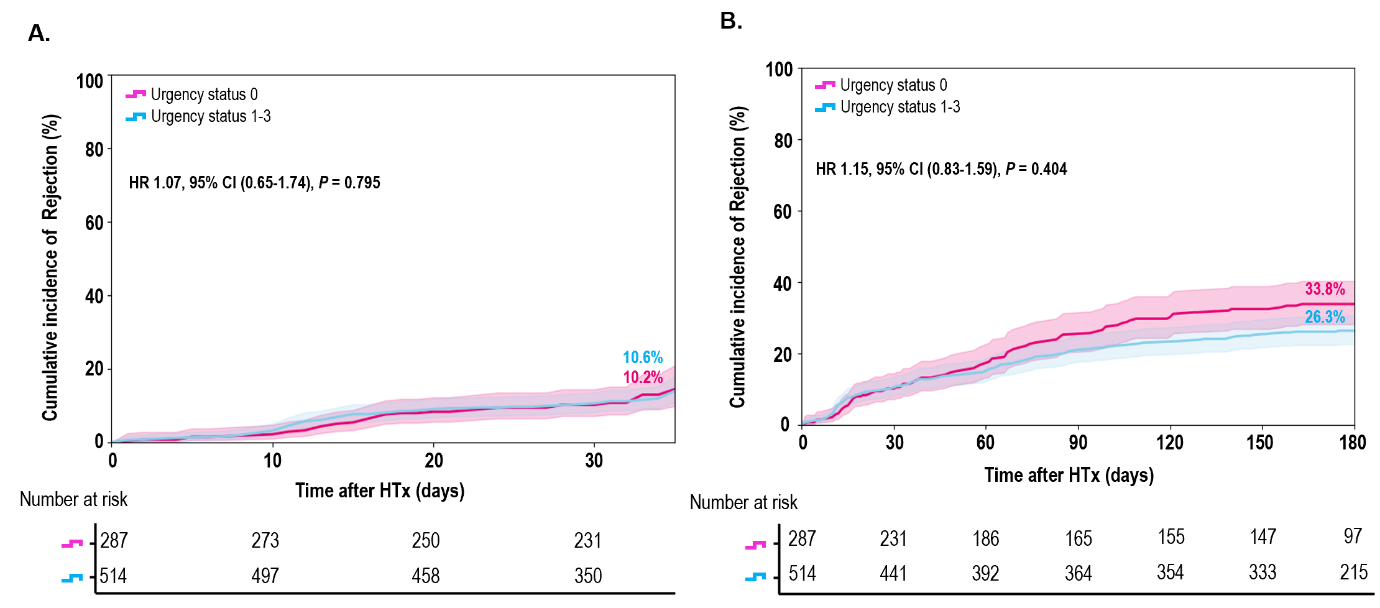


Cumulative incidence curves for acute rejection according to urgency status at the time of heart transplantation. (A) During the first month post-transplant, the incidence of rejection was comparable between Status 0 and Status 1ￚ3 recipients (HR 1.07, 95% CI 0.65–1.74, *P* = 0.795). (B) At 6 months post-transplant, rejection risk remained similar between the groups (HR 1.15, 95% CI 0.83–1.59, *P* = 0.404). Shaded areas represent 95% confidence intervals.

Abbreviations: HR, hazard ratio; CI, confidence interval

**Figure S3. Sensitivity analysis of clinical outcomes according to Urgency Status using KONOS criteria before the 2017 revision**

**A. Cumulative Hazard Curves for Mortality**


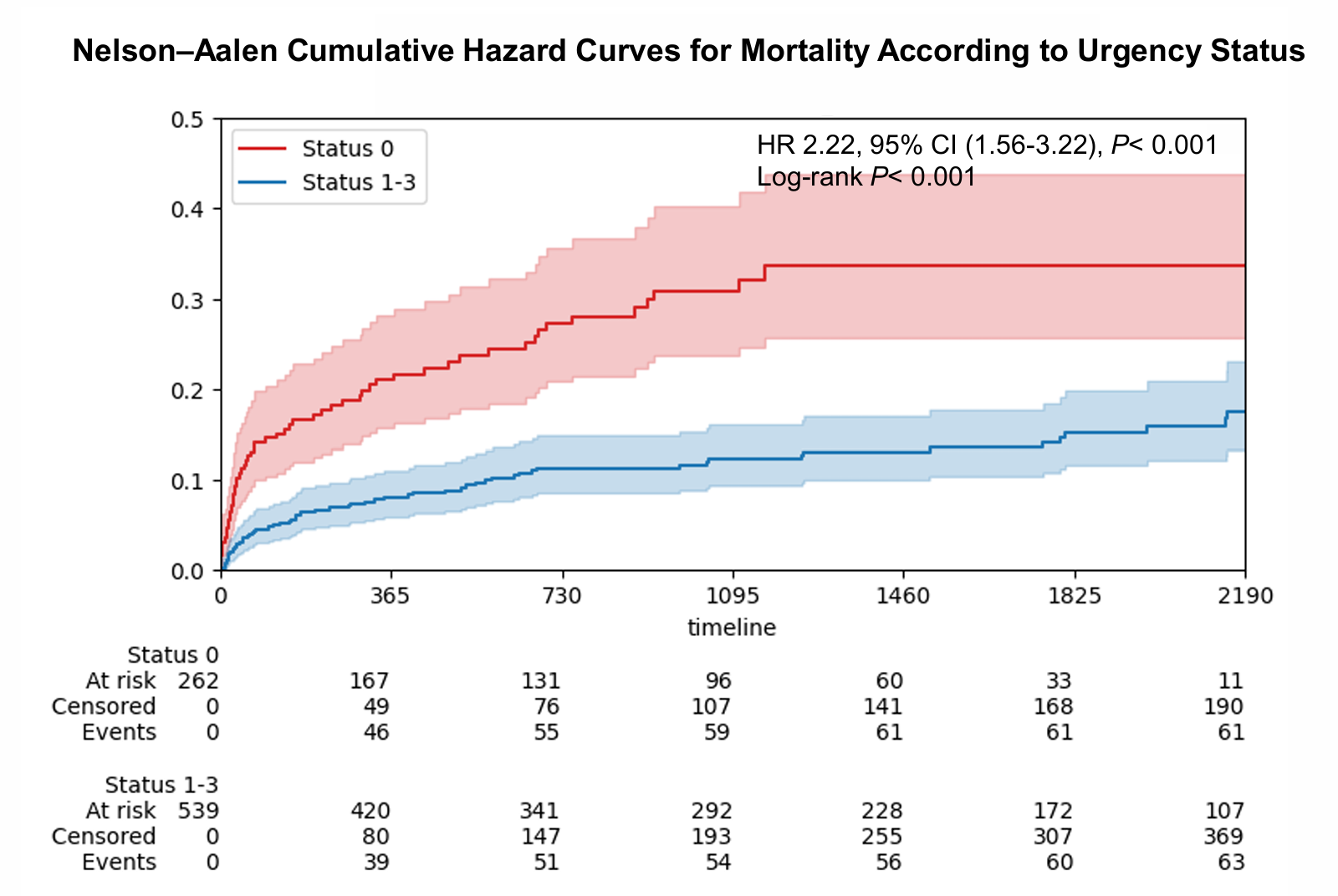


**B. Distribution of causes of death**
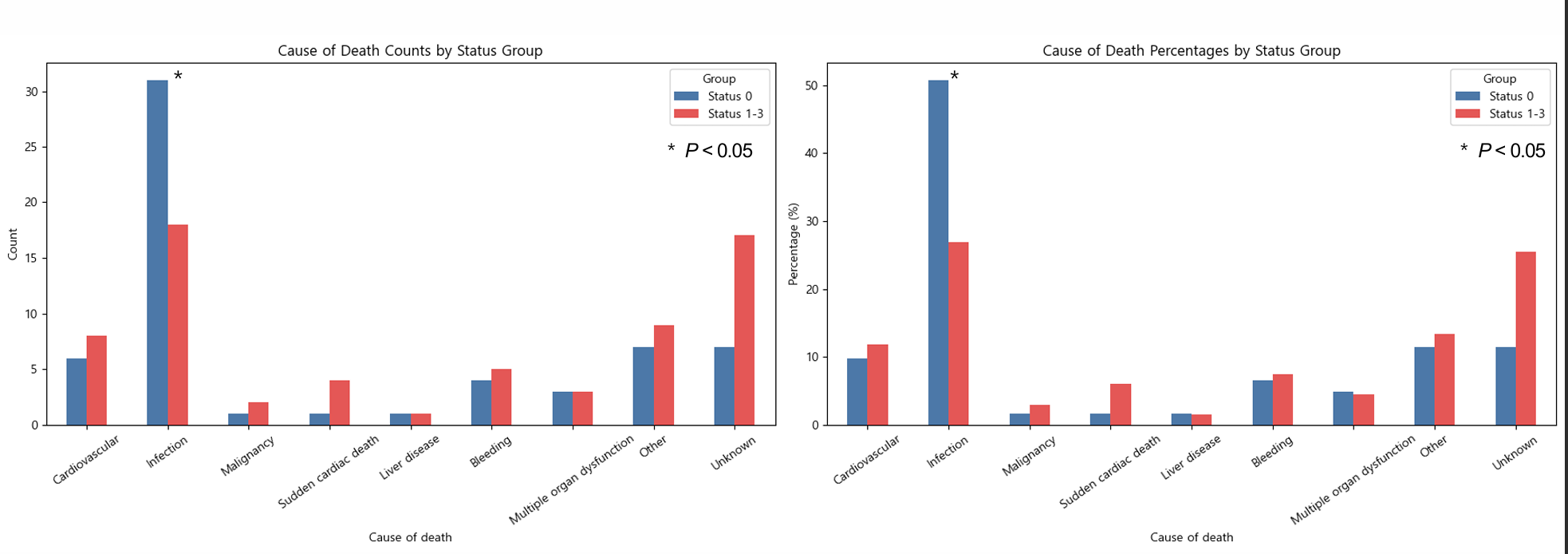


**C. Status-stratified predictors of all-cause mortality after heart transplantation**
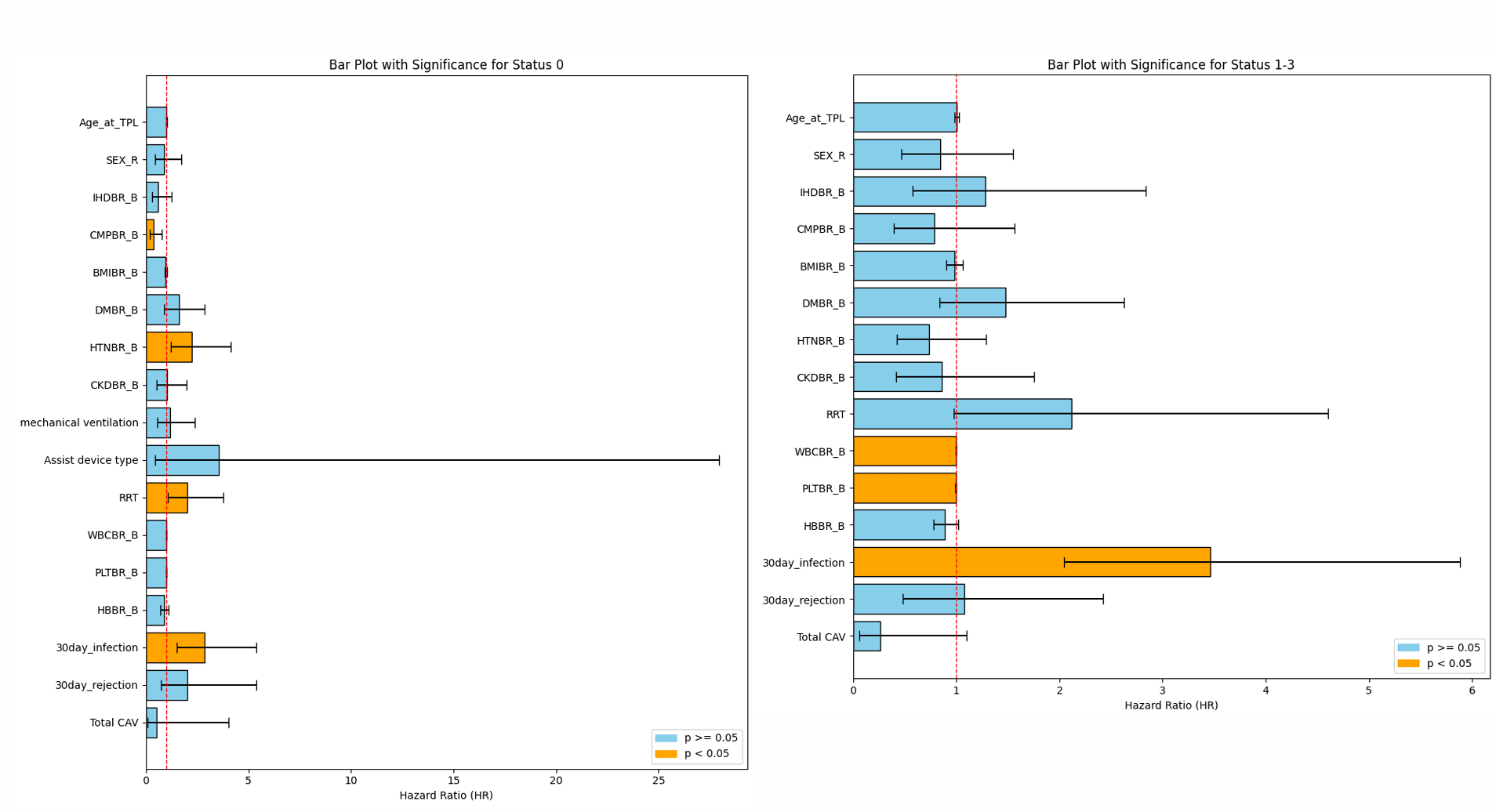


**D. Cumulative incidence of post-transplant infection at 30-, 90-, and 180-days
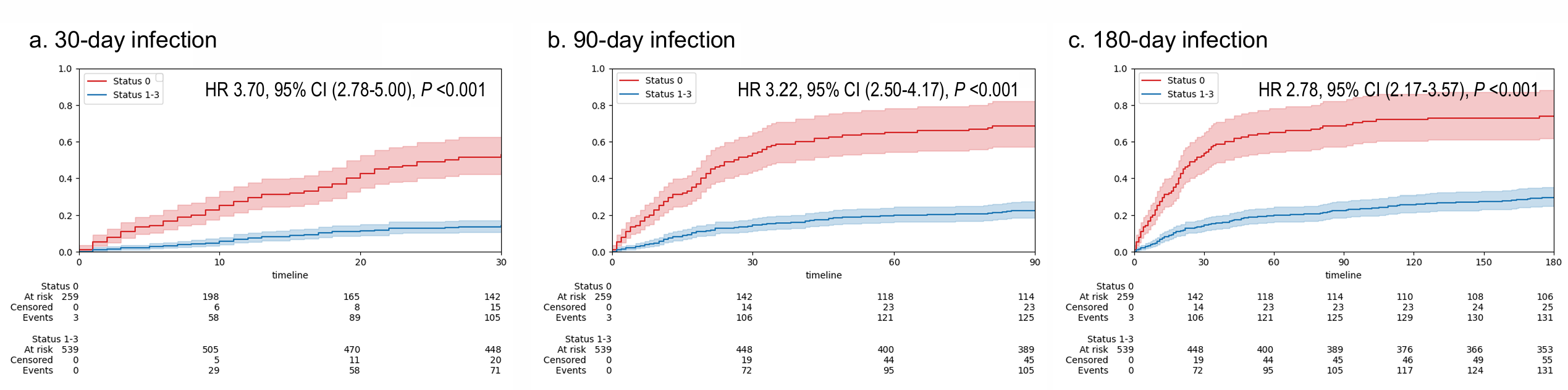
**

**E. Cumulative incidence of acute rejection at 30, 90, and 180 days**

**
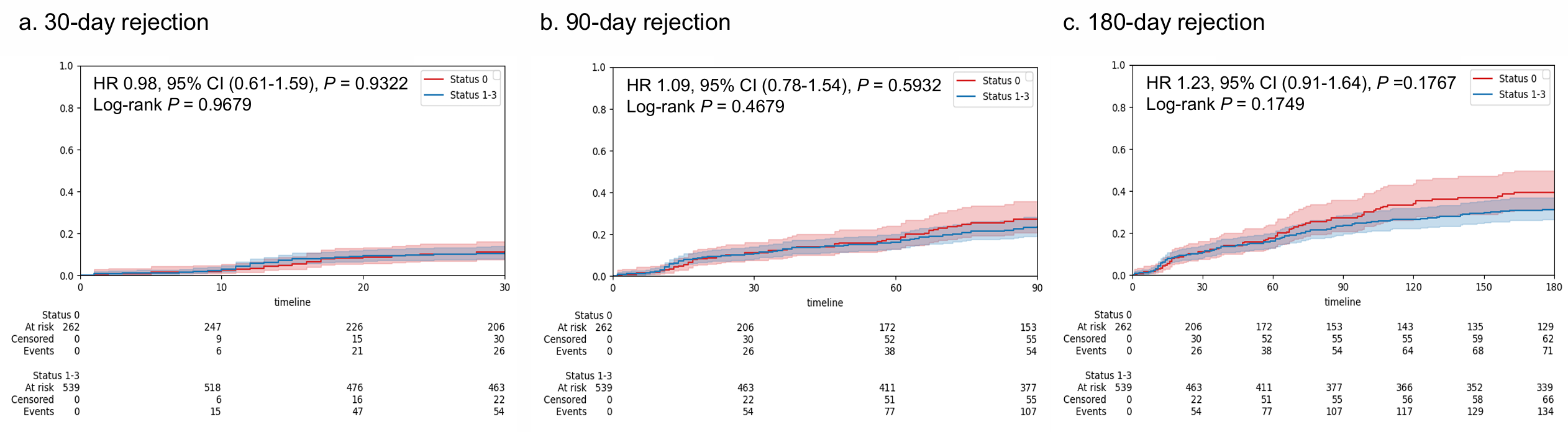
**

**F. Cumulative incidence of cardiac allograft vasculopathy**


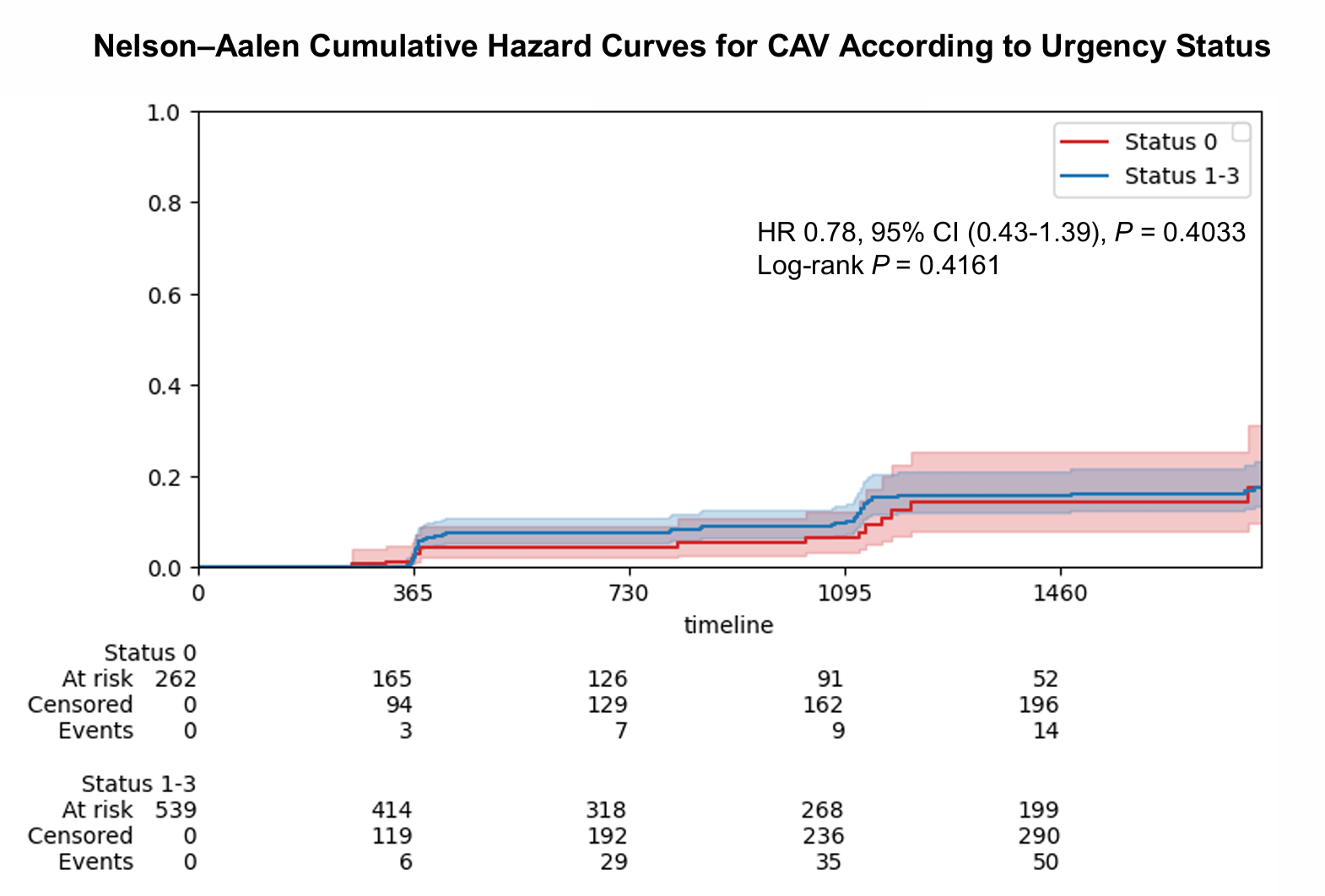


Clinical outcomes were compared between Status 0 and Status 1–3 recipients after applying the pre-revision KONOS urgency classification criteria. (A) Cumulative hazard curves for all-cause mortality according to urgency status. (B) Distribution of causes of death in each urgency status group. (C) Status-stratified Cox regression analysis of predictors of all-cause mortality after heart transplantation. (D) Cumulative incidence of post-transplant infection at 30, 90, and 180 days. (E) Cumulative incidence of acute rejection at 30, 90, and 180 days. (F) Cumulative incidence of cardiac allograft vasculopathy. Hazard ratios (HR) and 95% confidence intervals (Cl) are shown where applicable. Asterisks indicate statistically significant between-group differences.

Abbreviations: TPL, transplantation; IHD_BR, ischemic heart disease as the primary indication for heart transplantation; CMP_BR, cardiomyopathy as the primary indication for heart transplantation; BMI_BR, baseline body mass index at transplantation; DM_BR, diabetes mellitus at transplantation; CKD_BR, chronic kidney disease at transplantation; RRT, renal replacement therapy; WBC_BR, baseline white blood cell count at transplantation; PLT_BR, baseline platelet count at transplantation; HB_BR, baseline hemoglobin level at transplantation; CAV, cardiac allograft vasculopathy

**Figure S4. Temporal impact of infection, rejection, and cardiac allograft vasculopathy on all-cause mortality after heart transplantation**


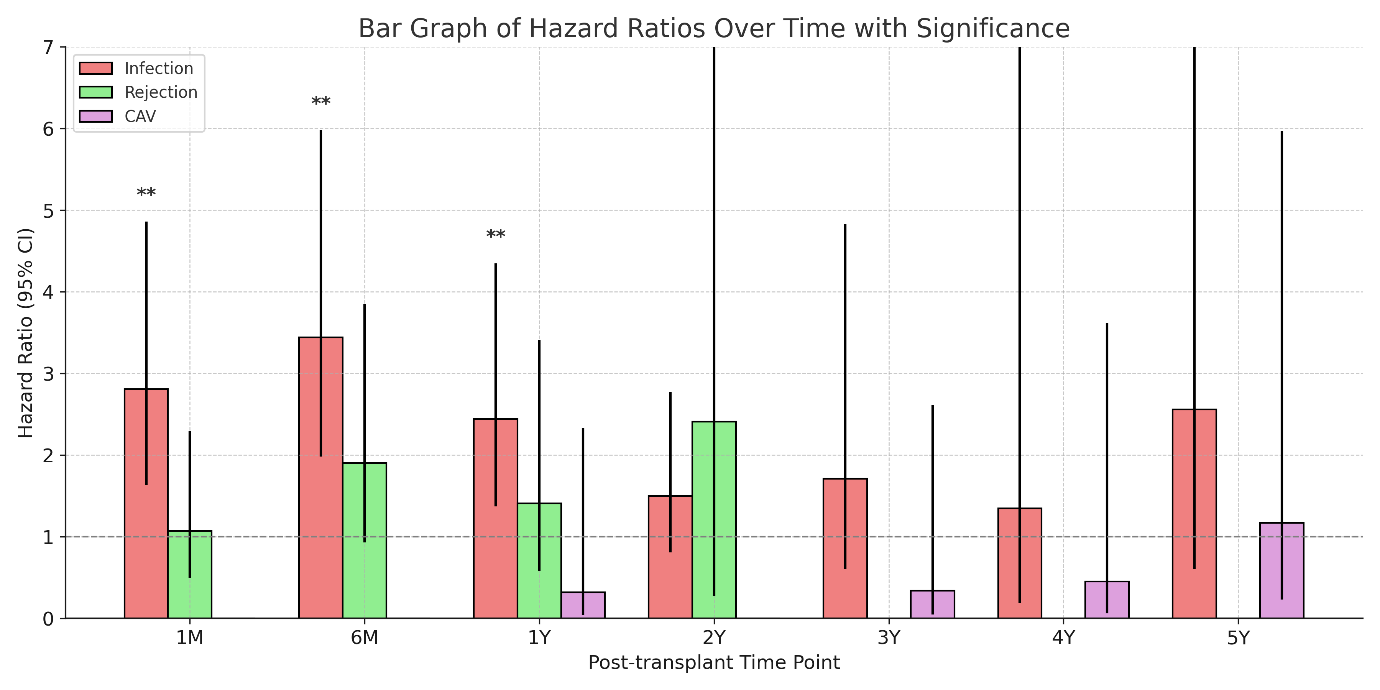


Bar graph shows the time-dependent association between infection (red), rejection (green), and cardiac allograft vasculopathy (purple) with all-cause mortality at various post-transplant time intervals. Each bar represents the hazard ratio (HR) with corresponding 95% confidence intervals across specified time windows. Asterisks indicate statistically significant associations (**P* < 0.05, ** *P* < 0.01, *** *P* < 0.01). N/A indicates insufficient events to calculate reliable estimates in that time interval.

**Figure S5. Mediation analysis assessing the role of post-transplant infection in the relationship between urgency status and all-cause mortality**


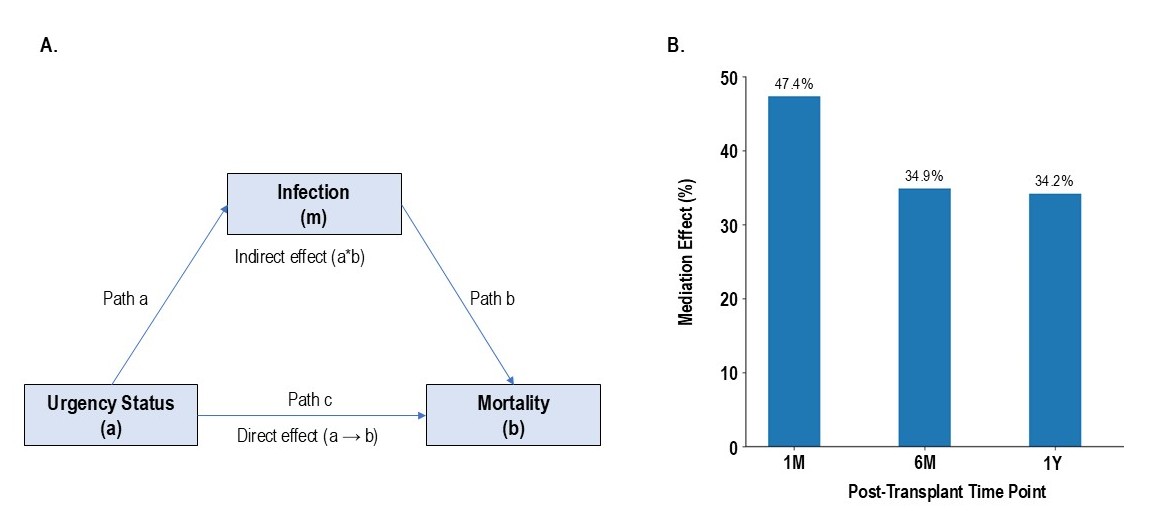


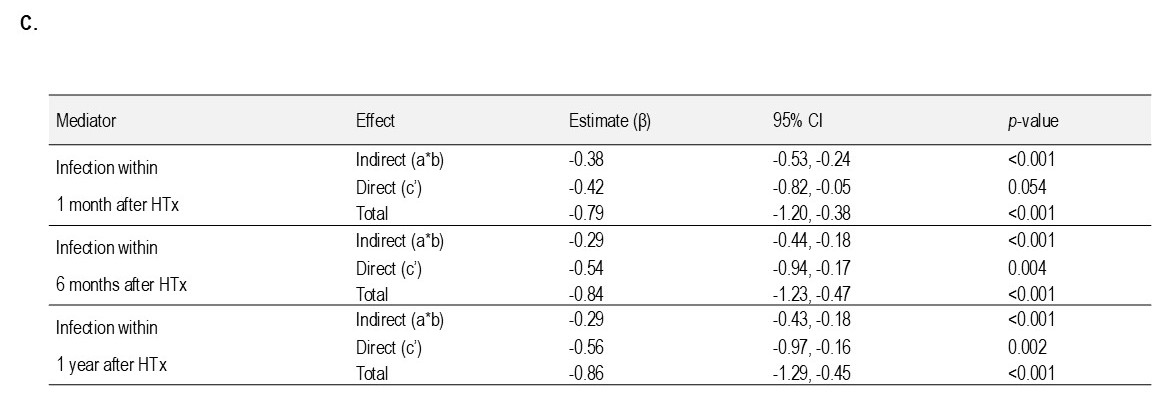


(A) Proposed mediational model depicting direct (a → b), indirect (a*b), and total effects (direct + indirect effect) of Urgency Status on mortality. Urgency Status as the independent variable (a), post-transplant infection at 1 month, 6 months, or 1 year as the mediator (m), and all-cause mortality as the dependent variable (b). The indirect effect (a*b) represents the impact of the urgency status group on mortality, which is mediated through infection. The direct effect (a → b) represents the influence of the urgency status group on mortality without considering infection as a mediator. The mediation analysis was performed using the bootstrap method with 1000 iterations.

(B) The bar graph illustrates the mediation effect at 1-month, 6-month, and 1-year intervals following heart transplantation. The magnitude of the mediation effect represents the proportion of the indirect effect relative to the total effect in the mediation model and is calculated using the following formula.

$Mediation Magnitude (\%)=\frac{Indirect effect (a*b)}{Total effect (Indirect+Direct effect)}$ x 100

(C) The table represents the mediation effects of post-transplant infections occurring within 1 month, 6 months, and 1 year after HTx on the relationship between Urgency Status at transplant and all-cause mortality. The total effect (c) is decomposed into indirect effects (a*b), mediated by infection, and direct effects (c’), not mediated by infection. Estimates (β), 95% confidence intervals (CI), and *p*-values are shown.

**Figure S6. Comparison of the use of pre- and post-transplant supportive interventions and mechanical circulatory support devices by urgency status**

**
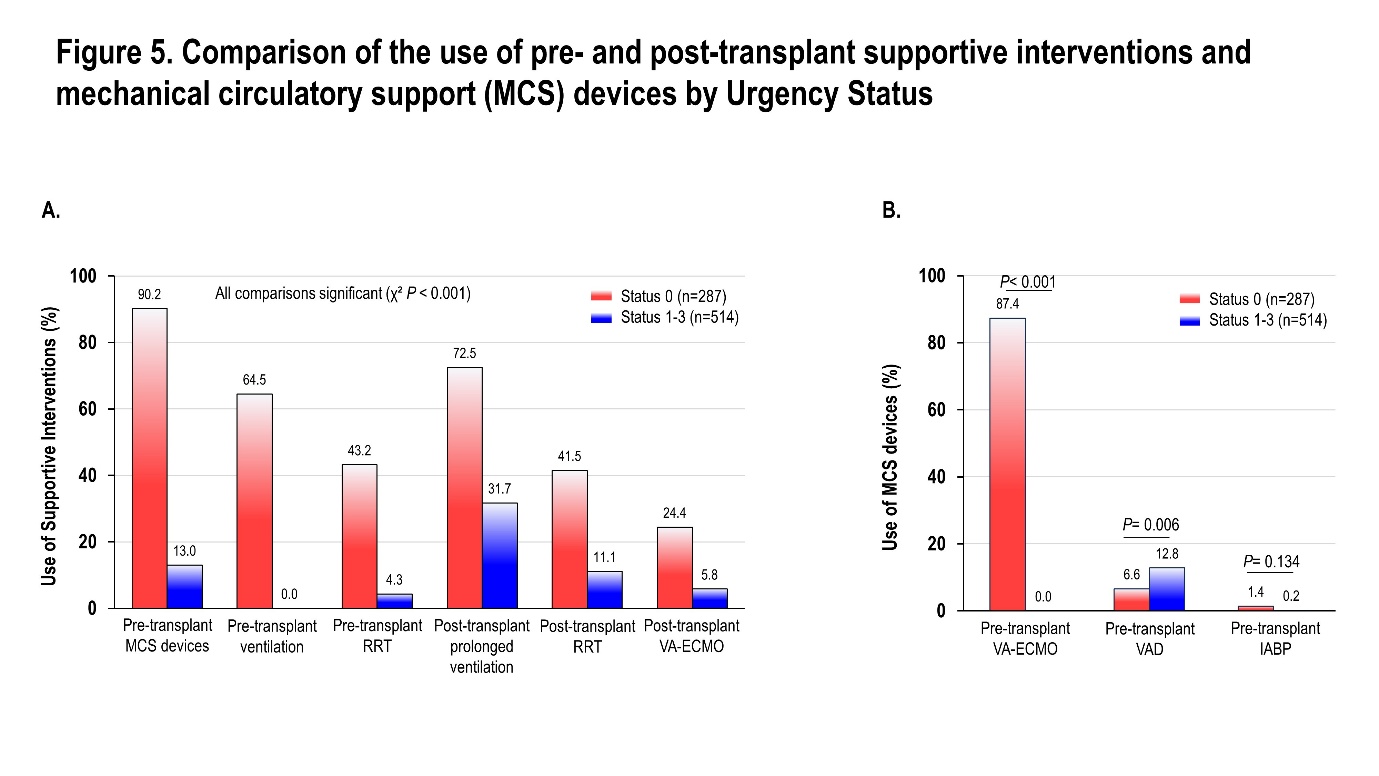
**

Bar graphs showing the differences in the use of (A) pre- and post-transplant supportive interventions and (B) MCS devices between recipients with Status 0 and those with Status 1–3.

Abbreviations: MCS, mechanical circulatory support; RRT, renal replacement therapy; VA-ECMO, venoarterial extracorporeal membrane oxygenation; VAD, ventricular assist device; IABP, intra-aortic balloon pump

**Figure S7. Pre-transplant risk factors and risk stratification model for 30-day post-transplant infection in Status 0 recipients.**

**A.**

**
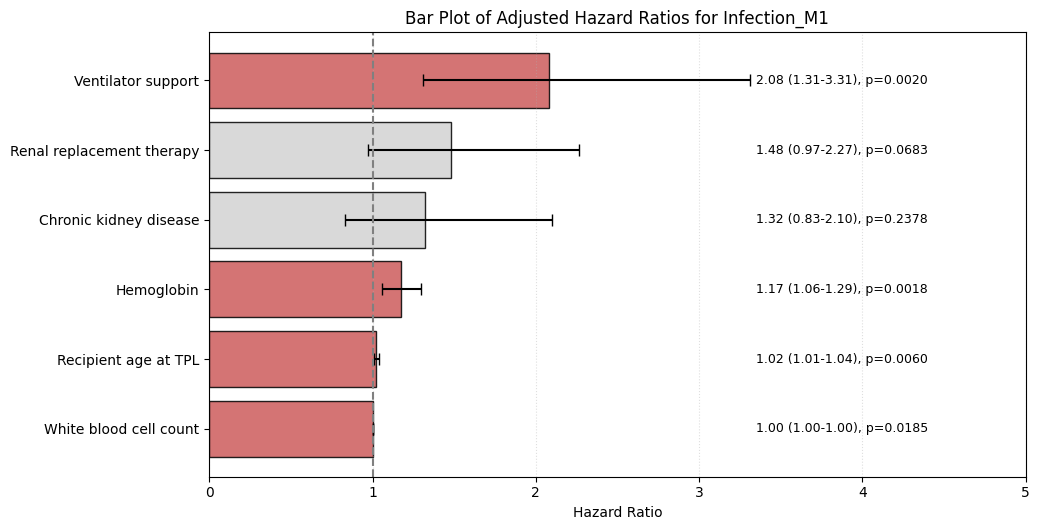
**

**B.**

**
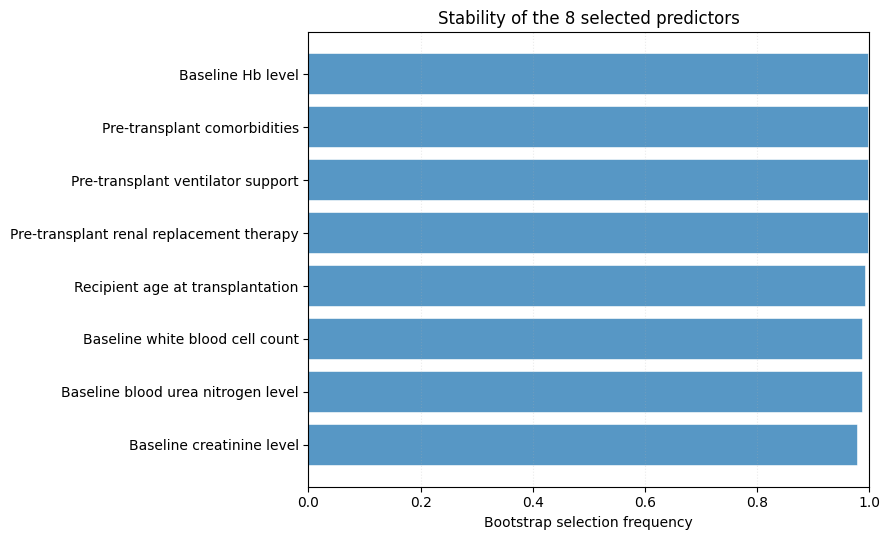
**

**C.**

**
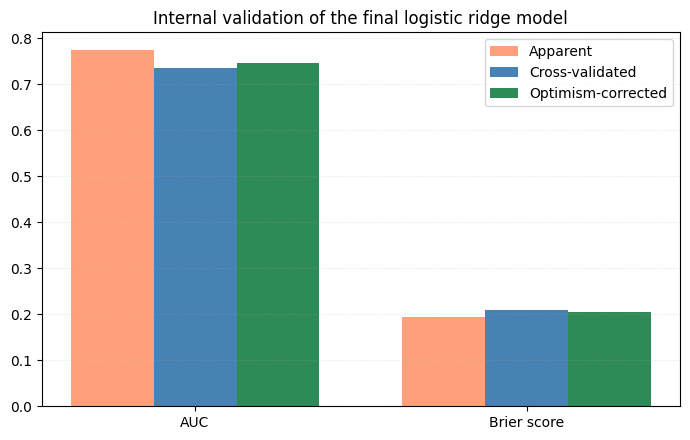
**

**D.**

**
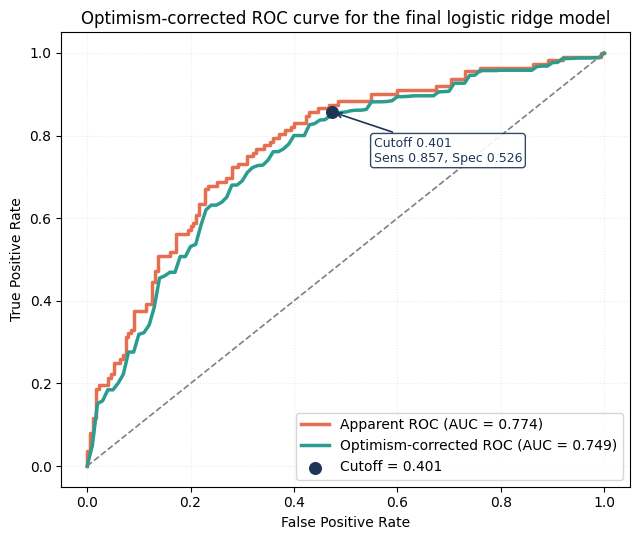
**

**E.**

**
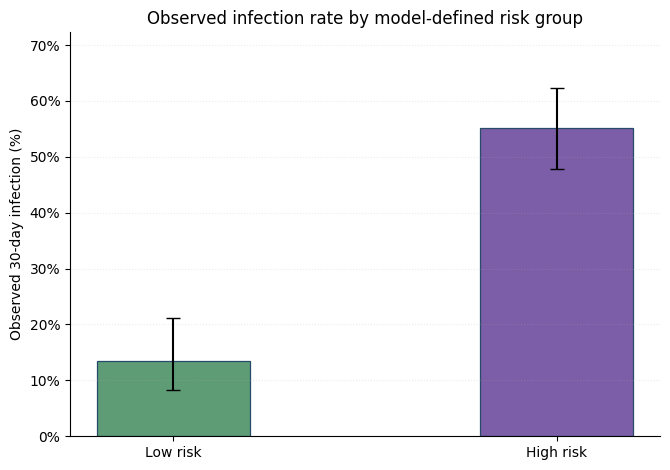
**

(A) Bar plot of adjusted hazard ratios from the final multivariable Cox model for 30-day post-transplant infection. Pre-transplant ventilator support, baseline hemoglobin level, recipient age at transplantation, and baseline white blood cell count were independently associated with infection risk. Error bars indicate 95% confidence intervals. (B) Bootstrap-based stability selection showing high reproducibility of the eight candidate predictors, with selection frequencies ranging from 98.0% to 100% across resamples. (C) Internal validation of the final logistic ridge model. The model showed stable discrimination and calibration after 5-fold cross-validation and bootstrap optimism correction, with an apparent AUC of 0.774, cross-validated AUC of 0.737, optimism-corrected AUC of 0.747, and optimism-corrected Brier score of 0.206. (D) Bootstrap optimism-corrected ROC curve showing internally validated model discrimination. The optimism-corrected AUC was 0.749, and the Youden index–based optimal cutoff was 0.401, yielding a sensitivity of 0.857 and specificity of 0.526. (E) Risk stratification using the Youden index–derived cutoff of 0.401, showing distinct observed 35-day infection rates between the low-risk and high-risk groups (13.5% vs. 55.1%).

Abbreviations: Infection_M1, post-transplant infection within 1 month after heart transplantation; TPL, transplantation; HB, hemoglobin level; ROC, receiver operating characteristic; AUC, area under the curve

**Figure S8. Impact of post-transplant ventilation duration on early post-transplant infection risk by urgency status**

**
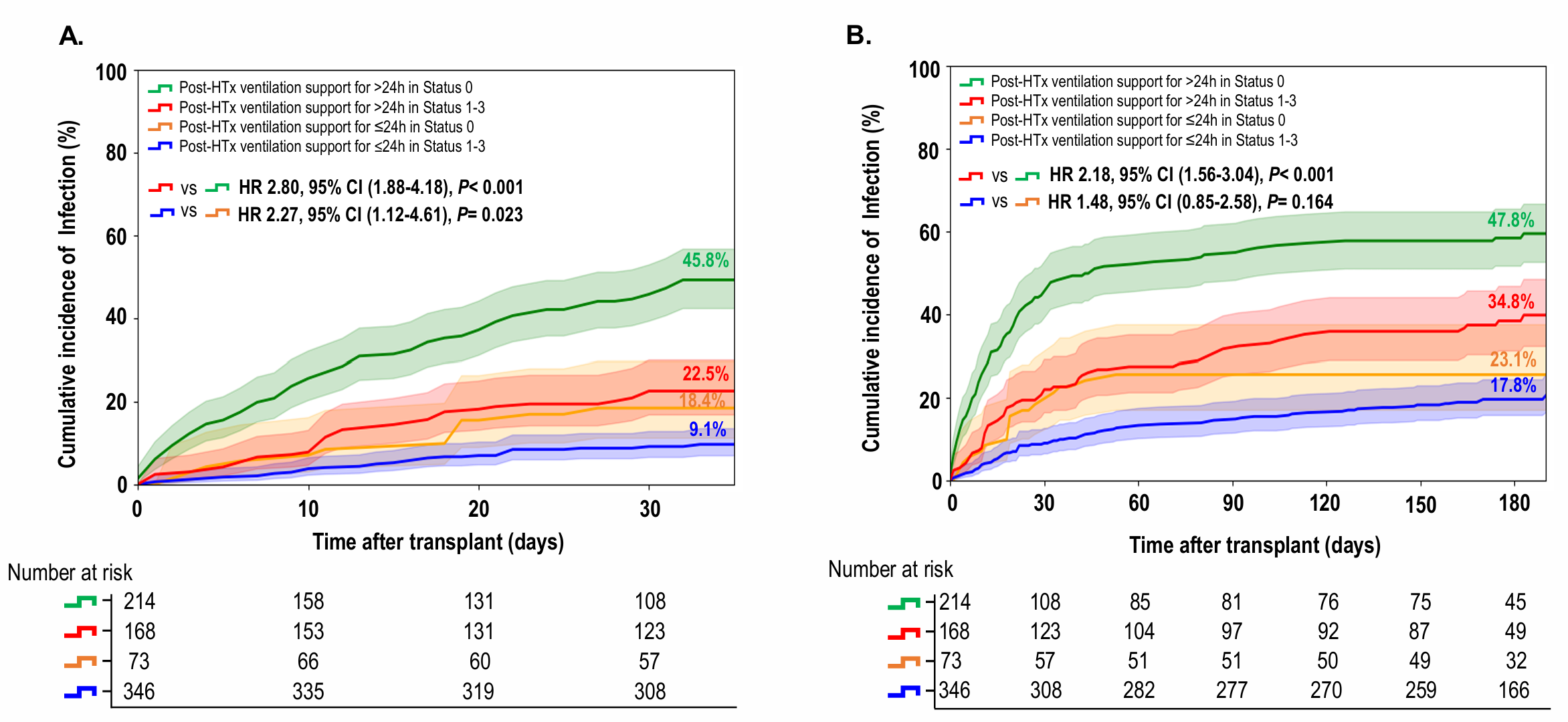
**

The Kaplan–Meier curves showing the cumulative incidence of (A) post-transplant infection and (B) rejection at 6 months post-transplant based on urgency status and the duration of post-transplant mechanical ventilation.

Abbreviations: HTx, heart transplantation; HR, hazard ratio; CI, confidence interval

**Figure S9. Impact of steroid tapering on early post-transplant infection and rejection risk by urgency status**

**
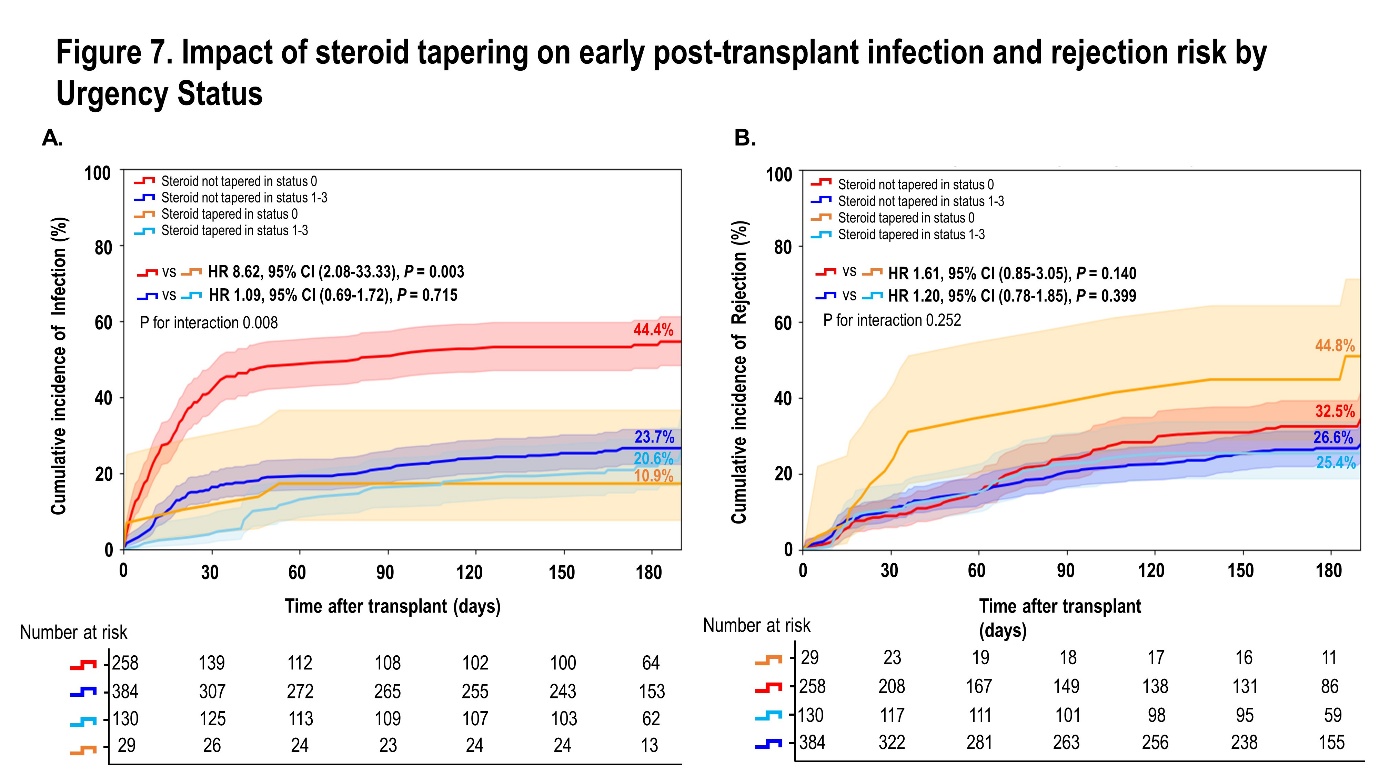
**

Kaplan–Meier curves showing the cumulative incidence of (A) post-transplant infection and (B) rejection at 6 months, comparing the results based on urgency status and steroid tapering status.

Abbreviations: HR, hazard ratio; CI, confidence interval
